# DeepFun: a deep learning sequence-based model to decipher non-coding variant effect in a tissue- and cell type-specific manner

**DOI:** 10.1093/nar/gkab429

**Published:** 2021-05-28

**Authors:** Guangsheng Pei, Ruifeng Hu, Peilin Jia, Zhongming Zhao

**Affiliations:** Center for Precision Health, School of Biomedical Informatics, The University of Texas Health Science Center at Houston, Houston, TX 77030, USA; Center for Precision Health, School of Biomedical Informatics, The University of Texas Health Science Center at Houston, Houston, TX 77030, USA; Center for Precision Health, School of Biomedical Informatics, The University of Texas Health Science Center at Houston, Houston, TX 77030, USA; Center for Precision Health, School of Biomedical Informatics, The University of Texas Health Science Center at Houston, Houston, TX 77030, USA; Human Genetics Center, School of Public Health, The University of Texas Health Science Center at Houston, Houston, TX 77030, USA; MD Anderson Cancer Center UTHealth Graduate School of Biomedical Sciences, Houston, TX 77030, USA

## Abstract

More than 90% of the genetic variants identified from genome-wide association studies (GWAS) are located in non-coding regions of the human genome. Here, we present a user-friendly web server, DeepFun (https://bioinfo.uth.edu/deepfun/), to assess the functional activity of non-coding genetic variants. This new server is built on a convolutional neural network (CNN) framework that has been extensively evaluated. Specifically, we collected chromatin profiles from ENCODE and Roadmap projects to construct the feature space, including 1548 DNase I accessibility, 1536 histone mark, and 4795 transcription factor binding profiles covering 225 tissues or cell types. With such comprehensive epigenomics annotations, DeepFun expands the functionality of existing non-coding variant prioritizing tools to provide a more specific functional assessment on non-coding variants in a tissue- and cell type-specific manner. By using the datasets from various GWAS studies, we conducted independent validations and demonstrated the functions of the DeepFun web server in predicting the effect of a non-coding variant in a specific tissue or cell type, as well as visualizing the potential motifs in the region around variants. We expect our server will be widely used in genetics, functional genomics, and disease studies.

## Graphical Abstract

**Graphical Abstract:**
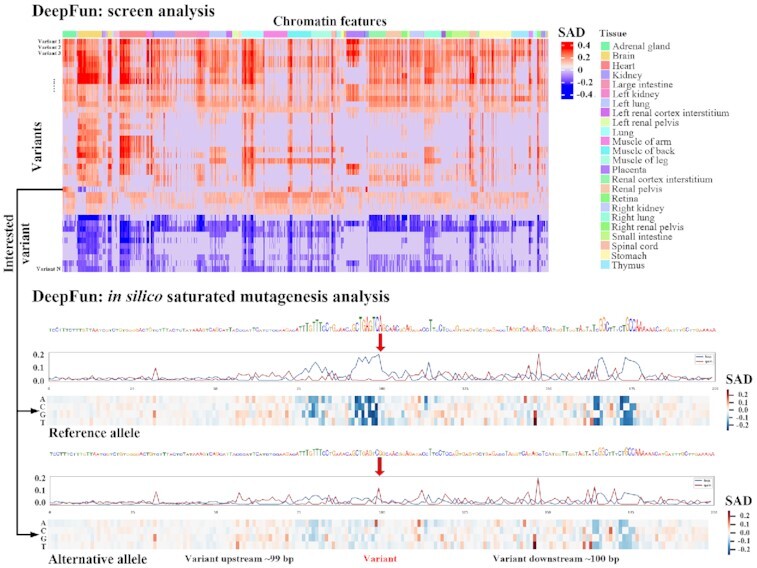
We implemented screen analysis and in silico saturated mutagenesis analysis in DeepFun webserver.

## INTRODUCTION

Functional interpretation of genetic variants from genome-wide association studies (GWAS) is critical to understanding the molecular mechanisms of complex diseases (1,2). This is because more than 90% of the genetic variants from GWAS are located in non-coding regions (3), even in gene deserts (4), making downstream analysis tremendously challenging. To elucidate the potential molecular function of non-coding variants, previous studies have shown that most GWAS-reported variants are significantly enriched in regulatory regions (5,6), such as DNA accessibility and transcription factor (TF) binding regions (7,8). Current studies of the function of non-coding variants often focus on the alteration of TF binding accessibility. For example, the single nucleotide polymorphism (SNP) rs1421085, which has two alleles T and C, has been reported to disrupt a conserved motif in *ARID5B* repressor gene. This disruption results in derepression of a potent preadipocyte enhancer and 2-fold *IRX3* and *IRX5* gene expression during early adipocyte differentiation (9). On the other hand, although all human tissues carry on common genetic information, interpretation of variants on regulatory elements remains a main challenge due to the distinct transcription regulatory programs across different tissues or cell types (10). Notably, it has been revealed that the disease-related variants only take effect in the relevant tissue or cell type. For example, some risk variants for psychiatric disorders tend to be in neuron-specific regulatory regions, while some of the risk variants for Alzheimer's disease are concentrated in microglial enhancers (11,12). To better illustrate the mechanisms of causal variants, there is a pressing need to prioritize them in a tissue- or cell type-specific manner (13,14).

The comprehensive functional annotation data from large projects such as the Encyclopedia of DNA Elements (ENCODE) project (15) and the Roadmap Epigenomics project (16) provides a unique opportunity to systematically assess the impact of all functional elements in the human genome towards tissue or cell type characteristics. The current-omics data has made it possible to evaluate tissue- or cell type-specific regulatory elements by using accessible chromatin, histone modification and TF binding intensities on various DNA sequences (10). These data enabled us to assign biochemical functions for 80% of the genome, in particular outside of the well-studied protein-coding regions (15). With these available extensive training data sets, many deep learning-based frameworks, including DeepBind (17), DeepSEA (18), Basset (7), DanQ (19), Basenji (20), DeFine (10), ExPecto (21) and Seqweaver (22), have been developed and shown remarkable advantages over the conventional machine learning methods, such as CADD (23), GWAVA (24) and FunSeq2 (25). Nevertheless, to explore novel architecture and algorithms, collecting more comprehensive annotation and chromatin profiling would help to improve predictive accuracy, scalability and robustness (20,21). In addition to the functional impact of non-coding variants in the genome, it is important to further investigate the potentially impacted genes or motifs around functional variants (10,18).

To address these challenges, we introduced DeepFun web server, a deep learning model for functional evaluation (DeepFun) of genetic variants and assessment of their effect in a cellular context at single-base resolution. Based on the increasing availability of epigenetics tracks from ENCODE and Roadmap, we constructed DeepFun models (26) by integrating 1548 DNase I accessibility, 1536 histone mark and 4795 transcription factor binding profiles. It is important to note that our model does not take any specific variant information into consideration. This feature in DeepFun enables the prediction of accessibility effects even for those that have never been observed previously (e.g. novel mutations in any studies and de novo mutations identified from family-based studies). Base on previous comprehensive evaluation (26), we implemented two functions in DeepFun webserver to predict the functional impact of any query variants. We demonstrated that DeepFun can not only effectively assess the functional impact of a non-coding variant and its impact in a tissue- and cell type-specific manner, but also visualize potential motifs in the region around the variant. The webserver is accompanied by detailed help pages. The results can be viewed in the browser and downloaded for further local analysis or record keeping. DeepFun, which is freely available at https://bioinfo.uth.edu/deepfun/, is a useful tool for prioritizing non-coding variants in the genome-scale based on our algorithm for functional impact assessment.

## MATERIALS AND METHODS

### DeepFun model training and functions

**Figure 1.**
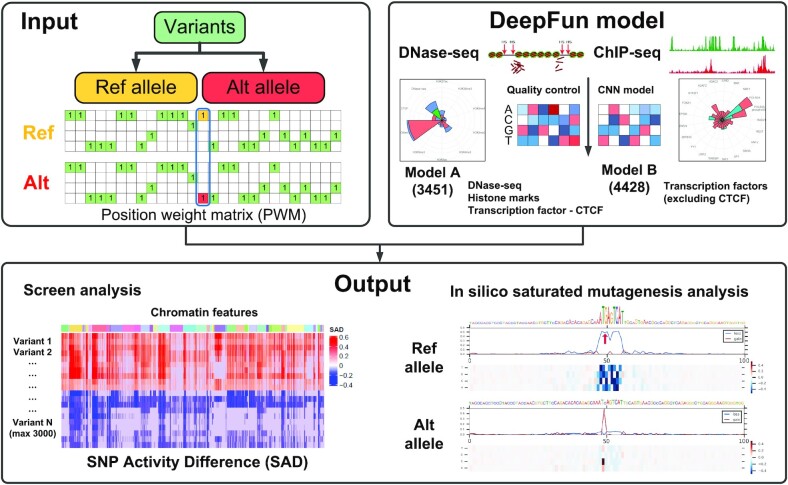
The framework of the DeepFun models and analysis. For each genetic variant, DeepFun considers its nearby 1000-bp genomic region as its context information, and then encodes it as one-hot code position weight matrix (PWM) to predict the accessibility or binding probability of sequences containing reference allele or alternative allele, respectively. To investigate the impact of variant, we implemented screen analysis and *in silico* saturated mutagenesis analysis.

DeepFun aims to predict the effects of genetic variants on a wide range of chromatin features, especially those located in non-coding regions. Figure 1 illustrates the framework of DeepFun web server. Briefly, we downloaded two types of epigenomics data from the ENCODE Project and the Roadmap Epigenomics Consortium: DNase-seq (DNA accessibility profiles) and ChIP-seq (include histone mark and transcription factor binding profiles), totaling 7879 samples (also called chromatin profiles). According to their functional category and completeness, we classified these assays into two models. Model A integrated 3451 samples, including all DNase-seq (1548), histone marks (1536) and the transcription factor CTCF (367) binding profiles. Model B integrated 4428 binding profiles for all the other TFs. After the removal of technical or biological replicates, DeepFun incorporates a total of 117 DNase-seq, 360 histone modification, and 795 TF binding profiles, representing the non-redundant number of assays.

For both datasets in models A and B, the downloaded annotation for peaks was reformatted as 1000 bp genomic intervals by extending 500 bp on each side of the midpoint of any narrow peaks reported in the original dataset, according to the Basset configuration (7). We then greedily merged peaks based on their distance to an adjacent peak, until no peaks overlapped by >200 bp (processed by the preprocess_features.py function). The center of the merged peak was determined as a weighted average of the midpoints of the merged peaks from the individual profile. These peaks were regarded as potential epigenomic active sites. Next, we applied an extended version of the Basset model (7) with default three convolutional layers, two fully connected hidden layers, and a fully connected sigmoid transformation layer to predict the peak accessibility or binding probability across different chromatin features. We randomly selected 80% of epigenomic active sites for training, 10% for validation, and 10% for testing, respectively. We used the area under receiver operating characteristic (AUROC) to evaluate the performance on validation and testing sets. The network training was stopped when the loss in the validation set did not decrease within 12 successive epochs of Bayesian optimization. By this measurement, we showed DeepFun achieved a median AUROC value of 0.933 over all DNase-seq assays, compared to 0.895 from the original Basset result (7). In addition, the area under precision-recall curve (AUPRC) was used to evaluate the model performance since the positive and negative datasets were imbalanced in size. The predicted effect for DNase-seq and histone mark assays had median AUPRC values of 0.544 and 0.354, compared to 0.042 and 0.025 from a random classifier. This resulted in a median AUPRC increase of 0.502 and 0.321 in DNase-seq and histone mark assays, respectively. More details of CNN model construction and performance for each feature can be found in our recent work (26).

The DeepFun model is designed to predict the functional impacts of sequence alterations at single-nucleotide resolution. However, DeepFun does not directly predict the impact of genetic variants. For each variant, DeepFun considers its neighboring 1000 bp region for context information, and then predicts the active (accessibility or binding) probability of sequence(s) containing either reference allele or alternative allele, respectively. To evaluate the impact of variant, we implemented the previously defined SNP Activity Difference (SAD) or relative log fold change of odds (log-odds) difference between the two alleles (26).

DeepFun implements two functions that can greatly facilitate the interpretation of genetic variants: the screen analysis (∼5 seconds per variant, up to 3000 variants per job) and the *in silico* saturated mutagenesis analysis (∼20 min, one variant per job). The screen analysis screens (by basset_sad.py function) for potential functional variants over all chromatin features rapidly, while the *in silico* saturated mutagenesis analysis (by basset_sat_vcf.py function) systematically scans along all potential single-nucleotide substitutions within 200 bp of the query variant to assess the effect of every base and prioritizes sequence features that are informative for a specific chromatin profile. The specific details of these two functions are provided on our website, the Help page, and also in the original method publication (26). The user-friendly web server DeepFun is available at https://bioinfo.uth.edu/deepfun/.

### Web server construction

The main functions of DeepFun were implemented in Python and Torch7 framework. The web server is hosted by a Linux server equipped with CentOS 7 and Apache (version 2.4) as the running environment. The server has four CPUs [Intel(R) Xeon(R) E5-2637 v3], 128 GB memory and 8TB hard disk to support computational tasks. DeepFun was designed and implemented in a standard Model-View-Controller (MVC) framework which is an architectural pattern widely used in modern web applications. The DeepFun web server contains three main logical components: Model, View, and Controller. On the backend, there are two well-trained CNN models for screen analysis and *in silico* saturated mutagenesis analysis, which execute the real computational task. The models take the input data delivered by the controller scripts and generate the results. In addition, DeepFun implements an email service to send notification messages to users regarding the running status of their submitted jobs. The email service is also part of our backend side function. Views are the frontend side interfaces for interactions with users. In DeepFun, the view pages are designed to guide users to input their data properly, show job running status, display feedback including practical errors, and present results when the job finishes successfully. These pages were implemented using HTML5, CSS, and JavaScript. To facilitate the different resolutions of users’ display screens, we used ‘Material Design for Bootstrap4 CSS library’ to build responsive web pages with fashion component designs. Ajax technology was used for calling the controller to submit jobs, retrieve data from the server-side, and update the information in views without refreshing the web page by utilizing the jQuery library. PHP was also used as an auxiliary function for data display. Controllers in our DeepFun are responsible for validating the input data and passing data between the frontend side views and the backend side models. The controllers were mainly written in the Python language (version 3.6). The mod_wsgi module was used for Apache to work with Python scripts. By utilizing the MVC framework, it provides a clear separation of logic making the testability to be frictionless. Our design allows us to extend functions and add plugins easily in the future. To avoid server overloading or task jams, we designed a queue strategy to organize the submitted jobs. Each user can submit as many jobs as necessary. DeepFun allows a maximum of 5 jobs running in parallel and can automatically deal with the jobs in a queue.

**Figure 2.**
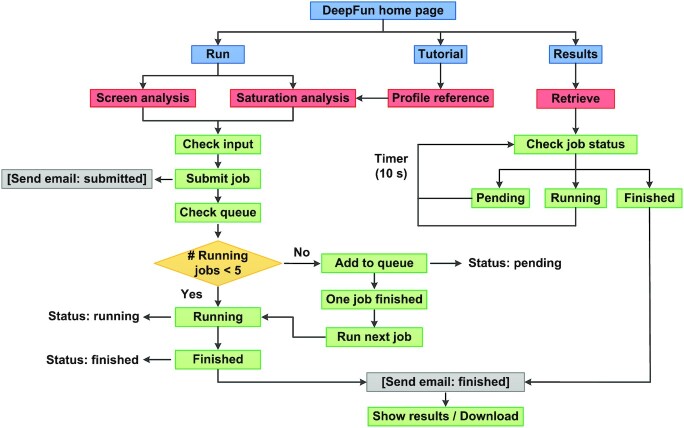
Overview of DeepFun web portal and workflow. DeepFun aims to predict the effects of genetic variants on a wide range of chromatin features based on deep convolutional neural networks (CNN). DeepFun provides two major functions: screen analysis and *in silico* saturated mutagenesis analysis. DeepFun can process all the submitted jobs automatically using a queue strategy.

**Figure 3.**
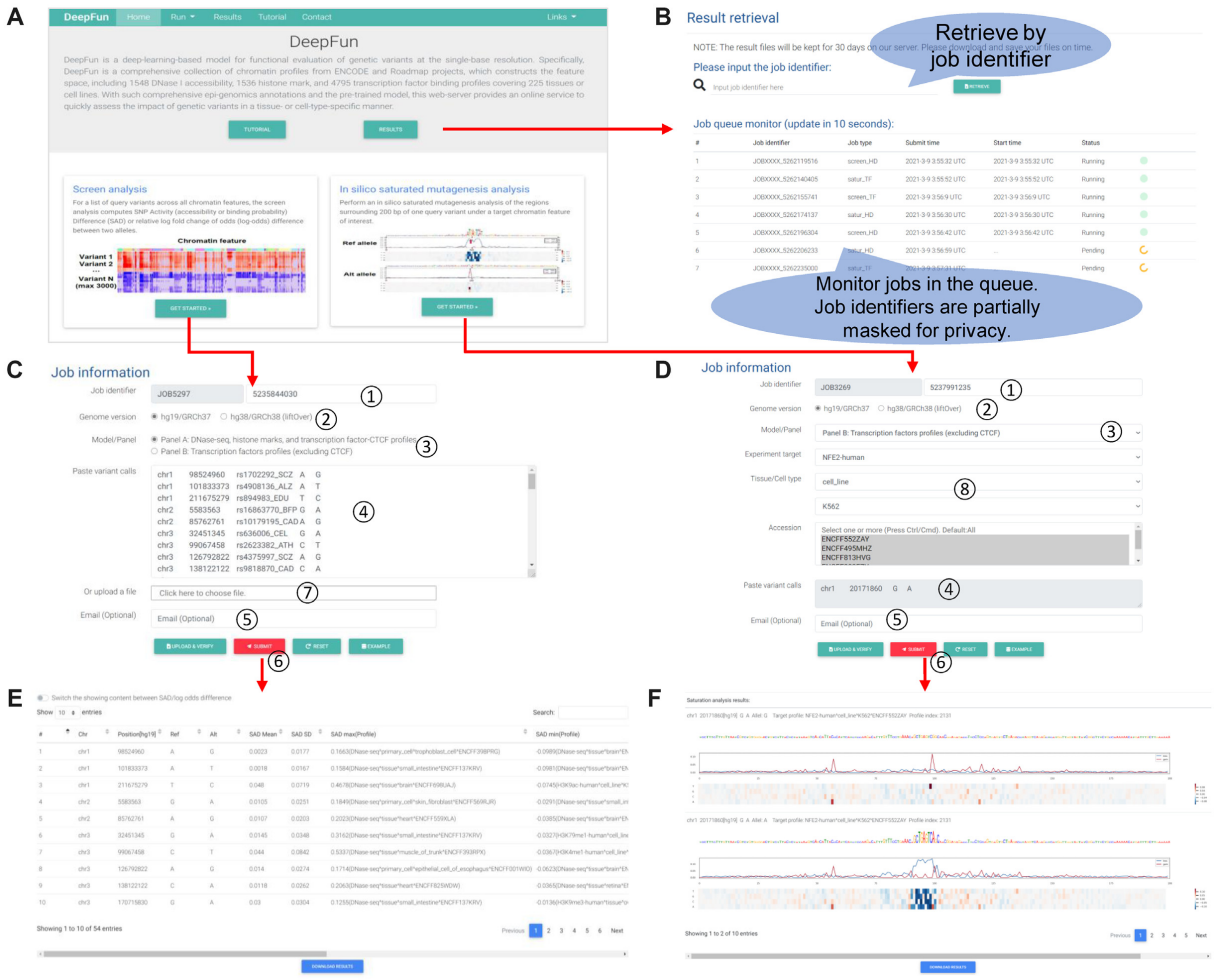
Web interfaces of the DeepFun server. (**A**) Home page of DeepFun. (**B**) Job monitoring and result retrieval page. (**C**) Input page of the screen analysis. (**D**) Output page of the screen analysis. (**E**) Input page of the *in silico* saturated mutagenesis analysis. (**F**) Output page of the Operation buttons for uploading *in silico* saturated mutagenesis analysis. Details in C and E are below. (1) Job identifier can be generated automatically or customized by the submitter. (2) Human genome reference assembly version: DeepFun supports the coordinates from the human genome version hg19/GRCh37 (default training) or hg38/GRCh38 (internal conversion to hg19/GRCh37). (3) Model selection: Model A or Model B. (4) Input box for variants. (5) Input box for email address. (6) Operation buttons for uploading and verifying inputs, submitting a job, resetting inputs and loading example data. (7) Function for uploading a file containing the variants in the required format. (8) Selection of user-specified profiles for *in silico* saturated mutagenesis analysis.

The workflow of the DeepFun server is illustrated in Figure 2. The coordinating web server interface is shown in Figure 3. On the home page (Figure 3A), users submit a job by selecting specific type of analysis and upload their data (Figure 3C and E). The web server will first conduct a preliminary check of the input data (e.g. correct formatting and nucleotides). If the input data is qualified, DeepFun sends a notification message of successful submission with the job identifier to the email address if provided. Before the job can be executed, DeepFun first checks the queue to find if there is a free slot (referred to a worker) available for the newly submitted job (Figure 2). Here, we name a worker as a CPU thread for executing the program. If a worker is available, DeepFun will run the new job and set the job status to ‘Running’. Otherwise, DeepFun adds the new job to the end of the queue and sets the status to ‘Pending’. After a job is submitted successfully, users will be directed to a page for monitoring the job status, where the status information is updated every 10 s until the job is finished. A message will be sent to the provided email address upon the job completion. Depending on the submitted job, the analysis may take for a while. In that case, users can close the DeepFun webpage while they should keep a record of the job identifier. Users can always go to the Results page to check any job status (Figure 3B), where DeepFun lists all the running and pending jobs. Users can monitor their jobs by searching the web site using the job identifier.

### Input

In both the screen analysis and the *in silico* saturated mutagenesis analysis (Figure 3C, E), users can query SNPs. The inputs can be in a 4-column format: chromosome, position, reference allele, and alternative allele, separated by space or tab. Each variant is in one line. The input file can be in the VCF-like format with five or more columns, where the first five columns are required, without a header line. These five columns contain information for chromosome, position, SNP ID, the reference allele, and the alternate allele. For the screen analysis, DeepFun accepts up to 3000 variants per job over either model A or model B. Users can paste the variants in the input box on the web page or upload a file that contains the variants following the formats as required. For *in silico* saturated mutagenesis analysis, DeepFun will only run the analysis on one variant each time over a group of experiment targets in a specific tissue or cell line, due to the time consuming of the very process.

Currently, we support input variants using the human genome version hg19/GRCh37 or hg38/GRCh38 (liftOver). DeepFun models were trained using profiling data based on hg19/GRCh37 coordinates. Thus, for input variants based on hg38/GRCh38 coordinates, the DeepFun server first applies the liftOver software (27) to convert hg38/GRCh38 coordinates to hg19/GRCh37. Variants that cannot be mapped to appropriate hg19/GRCh37 coordinates will be excluded from further analysis. Of note, DeepFun only accepts variants located on chromosomes 1 to 22 and X. Any variants located on the Y chromosome or mitochondrion genome will be automatically declined. This is consistent with GWAS study design and analysis, which has weaker power on the statistical association analysis for the variants on sex chromosomes, especially Y chromosome. In addition, when neither reference nor alternative allele can match to the reference genome, an error log file will be automatically generated to guide the user to remove aberrant variants or to check the correct genome version.

### Output

#### Screen analysis

To predict the effects of any input genetic variants on a wide range of chromatin features, DeepFun screen analysis will predict the activity (accessibility or binding) probabilities for each variant in its immediately neighboring sequence (1000 bp) carrying the reference and alternative allele over all chromatin features in the selected model. The predicted activity for both alleles ranges from 0 to 1. Then, the change of the predicted activity between sequence carrying reference and alternative alleles, which corresponds to the impact of the variant on accessibility or binding efficiency, is calculated over all chromatin features in the selected model. We implemented two measurements, SNP activity (accessibility or binding probability) difference (SAD) score, defined as: SAD = *P*(*alt*) − *P*(*ref*), and relative log fold change of odds difference between two alleles, defined as: log-odds difference = log(*P*(*alt*)/(1 − *P*(*alt*))) − log(*P*(*ref*)/(1 − *P*(*ref*))), where *ref* and *alt* represent the predicted activity probability for the reference allele (original sequence) and the alternative allele (mutated sequence), respectively. Both SAD and log-odds difference values reflect the degree of the functional impact of sequence alterations at single-nucleotide resolution. More details are described in our online tutorial.

Output pages are generated to facilitate a better visualization, screen potential functional variants, and present results for manuscript preparation. We demonstrate an output page for DeepFun screen analysis. As shown in Figure 3D, a summary page lists the average, standard deviation, maximum and minimum of SAD or log-odds difference values, as well as the most associated chromatin profiles for all the submitted variants across all chromatin features in a selected model. In addition, we present variants SAD heatmap summary to better demonstrate the impact of variants tissue- and cell type-specificity from the same assay. It is noted that only the top 50 variants with the highest maximum SAD values are displayed. For better custom figure, we deposit the heatmap plot code at Github https://github.com/bsml320/DeepFun.

To help users’ downstream analysis, we integrate hg19/GRCh37-based refGene annotation results derived from the ANNOVAR software (28) into the DeepFun screen analysis summary page. Users can download all the results to evaluate variant SAD or log-odd difference values over all chromatin features. For the variants based on hg38/GRCh38 coordinates, two additional files (the variants that can be mapped to hg19/GRCh37 and those variants are failed to map) are generated and reported to the user.

#### *In silico* saturated mutagenesis analysis

DeepFun performs ‘*in silico* saturated mutagenesis’ analysis to discover informative sequence features. Specifically, it will mutate every single base within 200 bp of the query variant and calculates the SAD change pattern in the target chromatin feature. As shown in Figure 3E, after determining the human genome assembly version, users need to specify target features of interests: Model/Panel → Experiment target →Tissue/Cell type → Accession. The *in silico* saturated mutagenesis analysis is computationally intensive and takes some time to compute. Therefore, we only accept one variant each time in the current version. We encourage users to conduct DeepFun screen analysis first to prioritize the target of interested chromatin features, for example, those feature(s) with absolute SAD > 0.1, before they perform *in silico* saturated mutagenesis analysis.

We demonstrate an example output page for DeepFun *in silico* saturated mutagenesis analysis. As shown in Figure 3F, for each profile that the user selects, two heat maps are displayed to show the effects (SAD values) of predicted accessibility or binding activity from mutation at every position on reference and alternative alleles, followed by an estimation of each nucleotide contribution to sequence's binding activity. Red color indicates the mutation would increase epigenetic signal, while blue color indicates decreased epigenetic signal. Users can download all figures in PDF format. Furthermore, for the user's convenience of downstream analysis, a table that includes the max gain and loss SAD values within 200 bp of the query variant (from −99 to 100 bp) is provided.

#### Job archiving

Once the job is submitted successfully, a unique job identifier, which can be customized partially by the user, is automatically assigned to the job. The job identifier is confidential and can be used to track job status and retrieve the results. Upon the completion of a job, all the result files will be zipped into one file, and a download button will be provided on the job monitoring page. The DeepFun server will keep all submitted jobs for 30 days. Within this time frame, user can freely access their results and download the zipped file to a local computer (Figure 3D, F). We also deposit DeepFun pre-trained models at Github https://github.com/bsml320/DeepFun in order to facilitate users to run the pre-trained models on their local hardware.

### Prior application and evaluation

#### Screen analysis

**Figure 4.**
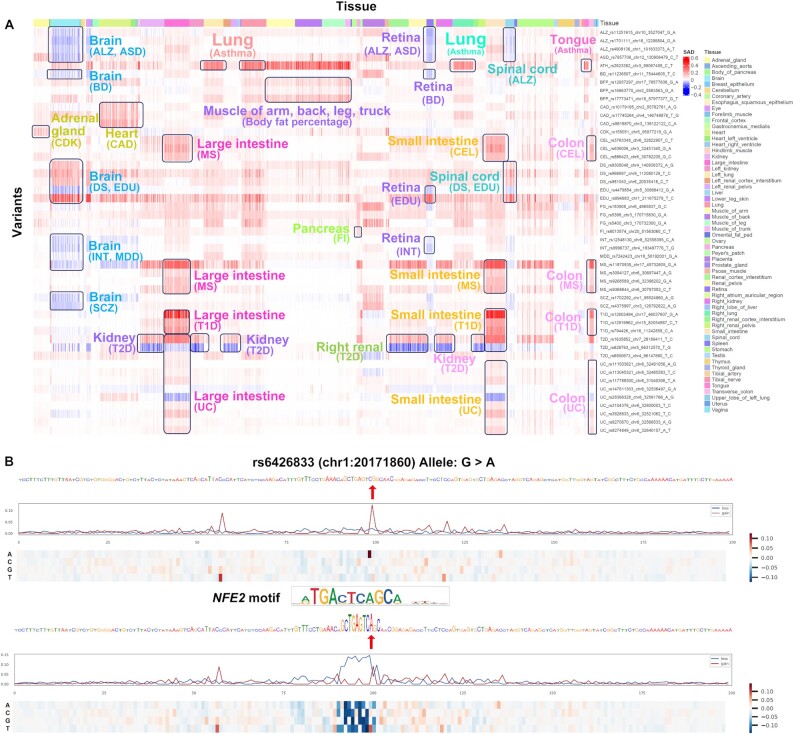
DeepFun evaluation and application. (**A**) Screen analysis of the multiple human complex traits and diseases with the associated SNPs over different DNA accessibility profiles. *X*-axis and *Y*-axis represent DNase accessibility profiles over different tissues and SNPs, respectively. (**B**) *In silico* saturated mutagenesis analysis of ulcerative colitis-associated SNP rs6426833 on transcription factor *NFE2* binding profile.

In our previous study, we have conducted several independent validations using the ClinVar genetic variants with benign, pathogenic and uncertain functions (29), *de novo* mutations in autism spectrum disorder (ASD) cohort from the Simons Simplex Collection (SSC) (30). Furthermore, we demonstrated DeepFun model could refine the significant GWAS associations to identify regulatory loci from background signals (26). Here, we further demonstrate DeepFun can predict non-coding variant's functional impact in a tissue- and cell type-specific manner. As shown in Figure 4A, most disease-related variants have higher SAD values in relevant tissues. For example, for most immune-related traits (celiac disease, multiple sclerosis, type 1 diabetes and ulcerative colitis), their associated risk loci have higher SAD values in intestines and colon. And for neurodegenerative and neuropsychiatric disease (Alzheimer's disease, autism spectrum disorder, depressive symptoms, education and schizophrenia), their risk loci have higher SAD values in brain and spinal cord. In addition, risk loci associated with body fat percentage had higher SAD values in muscle, coronary artery disease in heart and ventricle, fasting glucose and fasting insulin in pancreas and placenta, and type 2 diabetes in kidney and renal (Details in Supplementary Table S1). As above, our results suggested that DeepFun could effectively predict the functional impact of variants in a tissue-specific manner, as most of them are consistent with the disease symptoms. Therefore, they supported our methods being reliable.

To demonstrate that the model with an increased size would have better performance, we compared the results above with DeepSEA model, by calculating their *Pearson correlation coefficient* (PCC) for their averaged SAD score. As shown in Supplementary Figure S1, we observed a significant positive correlation in CTCF (PCC = 0.883, *P*-value = 2.2 × 10^−16^) and DNase-seq (PCC = 0.751, *P*-value = 1.46 × 10^−10^) assays between DeepFun and DeepSEA models. Although the numbers of assays are extremely imbalance for other histone marks (e.g. 595 H3K4me3 binding profiles in DeepFun, but only 8 in DeepSEA model), DeepFun still demonstrated a moderately positive correlation with DeepSEA model. For example, H3K4me3 (PCC = 0.490, *P*-value = 2.28 × 10^−4^), H3K9ac (PCC = 0.451, *P*-value = 7.99 × 10^−4^). We further compared them in cell type-specific manner. As shown in Supplementary Figure S2, most top impacted variants showed strong overlapping degree between DeepFun and DeepSEA. However, only ∼30% of these variants have a maximum SAD > 0.1 within DeepSEA model's 125 DNA accessibility profiles. Therefore, the analysis might lose those functional variants because they might only function in specific tissue or cell type. As shown in Figure 4A, the increased number of novel epigenomic datasets in DeepFun web server will be a valuable resource to decipher variant effect in a tissue- and cell type-specific manner.

#### *In silico* saturated mutagenesis analysis

To recognize informative sequence features from chromatin profiles, *in silico* saturated mutagenesis approach by scanning along all potential single-nucleotide substitutions was integrated to DeepFun model to assess the effect of mutating every base and informative sequence features for a specific chromatin profiles effect prediction (7,18). To validate performance of DeepFun model, we take one SNP (rs6426833) associated with ulcerative colitis (31) on transcription factor *NFE2* binding profile as an example. We generated two heat maps to display the SAD change patterns around the variant (from upstream 99 to downstream 100 bp) for both reference (G) and alternative (A) alleles. As shown in Figure 4B, *in silico* saturated mutagenesis predicted binding activity statistics in A allele sequence (bottom figure) is highly consistent with relevance *NFE2* binding motif TGACTCAGCA from CIS-BP database (32). Any mutations around *NFE2* motif and flanking regions would result in a decreased predicted binding activity. In contrast, only rs6426833 mutation from A to G would result in binding activity increase (Figure 4B), while other mutations around rs6426833 may not impact SAD (see details in Supplementary Table S2). Therefore, *in silico* saturated mutagenesis analysis is an effective way to dissect potential impacted motifs around functional variants (7).

## CONCLUSION

In this study, we have collected the comprehensive epigenomic annotation in DeepFun model. The abundant annotation will serve as useful resources for further exploration of the functional roles of non-coding variants in tissue- and cell type-specific manner. We demonstrated that the incorporation of multiple tissues and cell types would be a valuable approach to decipher those functional variants in tissue- or cell type-specific manner. In addition, with the help of *in silico* saturated mutagenesis analysis, the DeepFun web server will be useful for interpreting potential target gene's motifs around functional variants. We hope our web server will be a valuable resource in GWAS downstream analysis (26) and also broadly non-coding variant evaluation on the functional impact (30,33).

There are several ways to improve DeepFun server in the future. First, recent efforts to describe the human epigenome in ENCODE 3 (34) and advancement of computational methodologies (35,36) have yielded thousands of novel epigenomic maps. Investigating functional impact of non-coding variants can be improved by training with the growing number of epigenomic datasets (37). Second, the performance of most CNN-based methods may strongly depend on initial convolution filters for captured motif information. DeepFun model has not considered long-range interactions, such as 3D DNA contact (20,37). Therefore, novel CNN architecture, algorithms and integrating with other epigenomic experimental data will further improve the model accuracy. Third, during the training process, we treated the ‘peaks’ as the binary format to represent the experimentally observed signal around each candidate ‘peak’ region. This procedure helps mitigate bias and noise caused by the quality of antibodies and sequencing depth, or other factors that may affect the quantitative signals. However, it has ignored the quantitative information. In future, we will explore appropriate normalization approach to eliminate technical bias, and then integrate quantitative epigenomic signals into our updated model (20). We will continue to develop DeepFun as above, and regularly maintain the web server by adding more data and functions.

## DATA AVAILABILITY

All the data generated or analyzed in this study is available from the authors upon request. We deposit DeepFun pre-trained models and downstream analysis scripts at Github https://github.com/bsml320/DeepFun.

## Supplementary Material

gkab429_Supplemental_File
