# Supplementary material for: DeepFun: a deep learning sequence-based model to decipher non-coding variant effect in a tissue- and cell type-specific manner
Source: Nucleic Acids Res. 2021 May 28;49(W1):W131–9. doi: 10.1093/nar/gkab429 (PMC8262726; doi:10.1093/nar/gkab429)
Supplement: gkab429_Supplemental_File [file gkab429_supplemental_file.pdf]

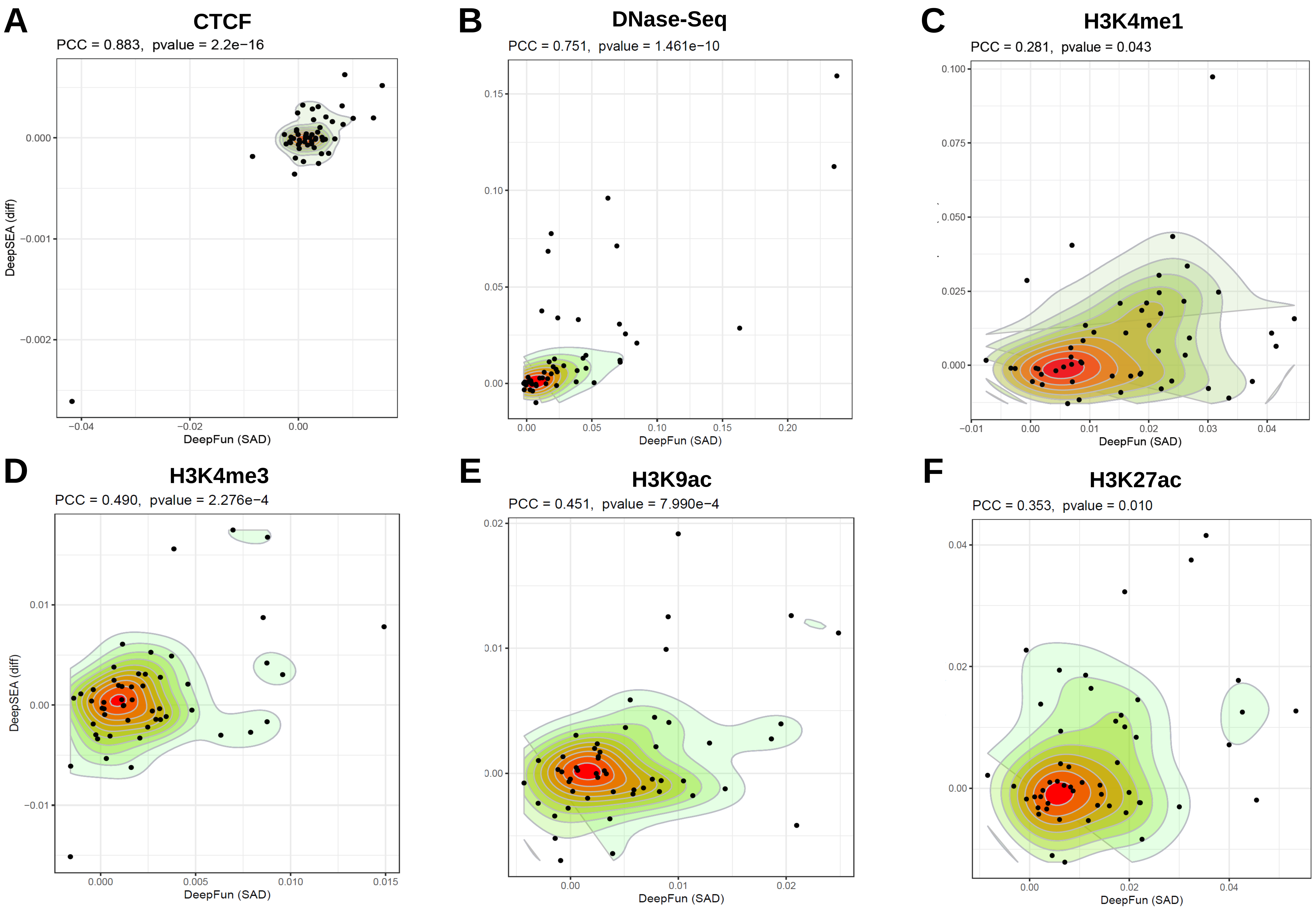

Supplementary Figure S1. The performance comparison between DeepFun and DeepSEA across different chromatin features. X-axis represents the averaged SAD score predicted by DeepFun model. Y-axis represents the averaged diff score predicted by DeepSEA model. **(A)** CTCF (DeepFun 207 replicates vs. DeepSEA 100 cell types). **(B)** DNase-seq (DeepFun 602 replicates in primary cells vs. DeepSEA 125 cell types). **(C)** H3K4me1 (DeepFun 76 replicates vs. DeepSEA 8 cell types). **(D)** H3K4me3 (DeepFun 595 replicates vs. DeepSEA 8 cell types). **(E)** H3K9ac (DeepFun 160 replicates vs. DeepSEA 7 cell types). **(F)** H3K27ac (DeepFun 371 replicates vs. DeepSEA 8 cell types).

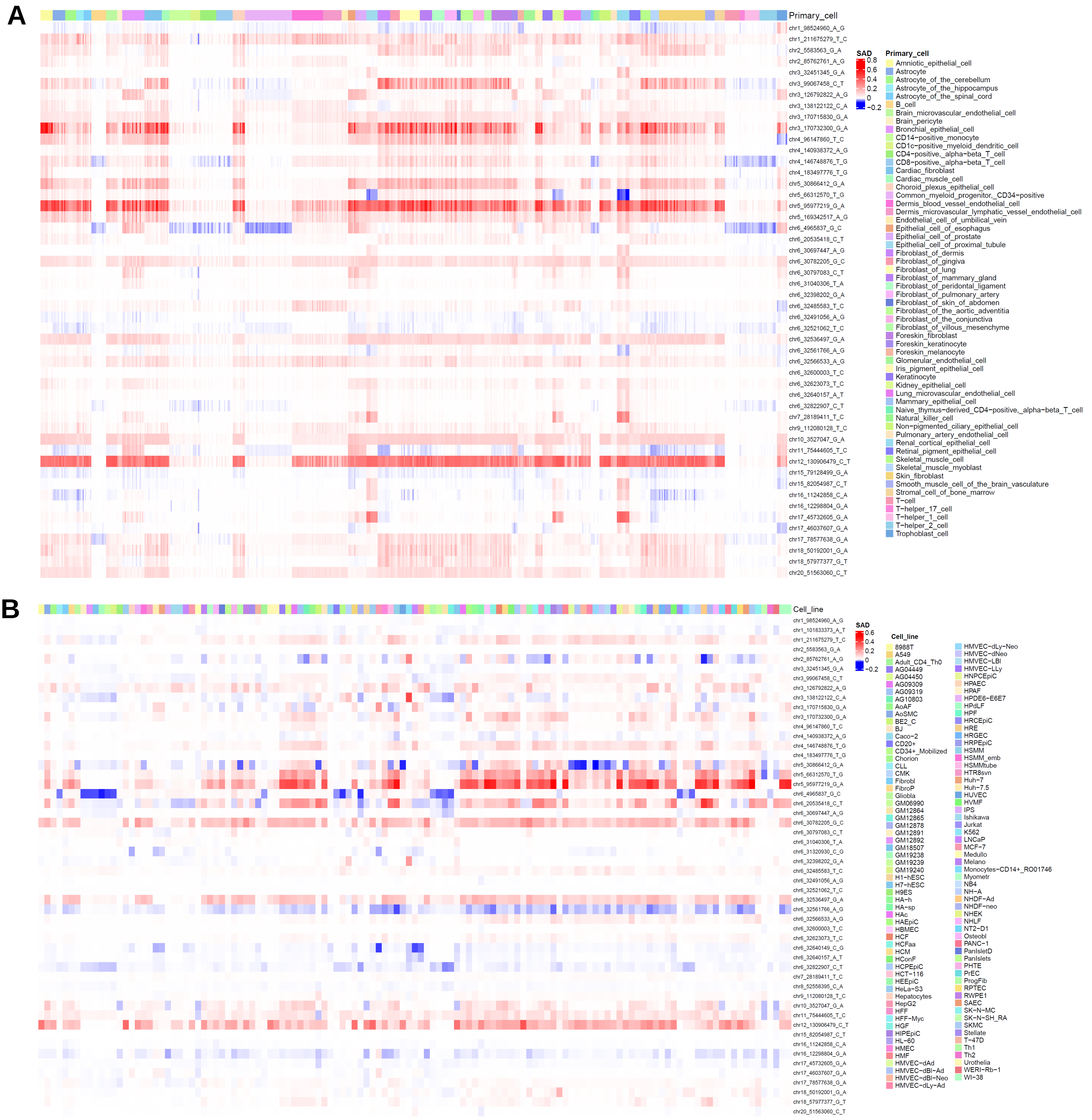

Supplementary Figure S2. The performance comparison between DeepFun and DeepSEA over different DNase-seq features. X-axis and Y-axis represent DNase accessibility profiles over different cell types and SNPs, respectively. **(A)** Predicted results of DeepFun using 602 DNase accessibility profiles over different primary cell types. **(B)** Predicted results of DeepSEA using 125 DNase accessibility profiles over different cell types.

Supplementary Table S1. Validation of human complex disease related variant's functional impact tissue-specificity in DNase-seq.

Chr	Pos	RS_ID	REF	ALT	Trait	Trait full name	Top1 relevant tissue	Top2 relevant tissue	Top3 relevant tissue	Top4 relevant tissue	Top5 relevant tissue
chr10	3527047	rs11251915	G	A	ALZ	Alzheimer’s disease	placenta	psoas_muscle	retina	pancreas	uterus
chr16	12298804	rs1701111	G	A	ALZ	Alzheimer’s disease	retina	brain	eye	Peyer's_patch	small_intestine
chr1	101833373	rs4908136	A	T	ALZ	Alzheimer’s disease	large_intestine	small_intestine	retina	transverse_colon	eye
chr12	130906479	rs7957706	C	T	ASD	Autism spectrum disorder	psoas_muscle	uterus	pancreas	transverse_colon	placenta
chr3	99067458	rs2623382	C	T	ATH	Asthma	hindlimb_muscle	muscle_of_trunk	muscle_of_leg	forelimb_muscle	muscle_of_back
chr11	75444605	rs11236507	T	C	BD	Bipolar disorder	placenta	body_of_pancreas	transverse_colon	pancreas	Peyer's_patch
chr17	78577638	rs12937297	G	A	BFP	Body fat percentage	hindlimb_muscle	tongue	heart_right_ventricle	muscle_of_trunk	heart
chr2	5583563	rs16863770	G	A	BFP	Body fat percentage	hindlimb_muscle	muscle_of_trunk	forelimb_muscle	lung	left_lung
chr18	57977377	rs17773471	G	T	BFP	Body fat percentage	hindlimb_muscle	muscle_of_trunk	forelimb_muscle	muscle_of_leg	brain
chr2	85762761	rs10179195	A	G	CAD	Coronary artery disease	heart_right_ventricle	heart_left_ventricle	heart	body_of_pancreas	adrenal_gland
chr4	146748876	rs17745264	T	G	CAD	Coronary artery disease	heart_right_ventricle	heart_left_ventricle	tongue	heart	lung
chr3	138122122	rs9818870	C	A	CAD	Coronary artery disease	heart_right_ventricle	heart_left_ventricle	heart	body_of_pancreas	testis
chr5	95977219	rs155051	G	A	CDK	Chronic kidney disease	psoas_muscle	pancreas	adrenal_gland	transverse_colon	uterus
chr6	32822907	rs3763348	C	T	CEL	Celiac disease	small_intestine	large_intestine	transverse_colon	eye	right_kidney
chr3	32451345	rs636006	G	A	CEL	Celiac disease	large_intestine	small_intestine	transverse_colon	body_of_pancreas	Peyer's_patch
chr6	30782205	rs886423	G	C	CEL	Celiac disease	transverse_colon	small_intestine	large_intestine	Peyer's_patch	placenta
chr4	140938372	rs9308048	A	G	DS	Depressive symptoms	brain	eye	muscle_of_trunk	hindlimb_muscle	tongue
chr9	112080128	rs966697	T	C	DS	Depressive symptoms	heart_right_ventricle	tongue	eye	body_of_pancreas	brain
chr6	20535418	rs981043	C	T	DS	Depressive symptoms	eye	brain	retina	tongue	spinal_cord
chr5	30866412	rs4479854	G	A	EDU	Education	retina	psoas_muscle	eye	placenta	brain
chr1	211675279	rs894983	T	C	EDU	Education	eye	retina	brain	tongue	heart_right_ventricle
chr6	4965837	rs183908	G	C	FG	Fasting glucose	placenta	body_of_pancreas	pancreas	transverse_colon	thyroid_gland
chr3	170715830	rs5398	G	A	FG	Fasting glucose	transverse_colon	pancreas	lung	left_lung	uterus
chr3	170732300	rs5400	G	A	FG	Fasting glucose	placenta	lung	left_lung	pancreas	right_lung
chr20	51563060	rs6013574	C	T	FI	Fasting insulin	pancreas	transverse_colon	body_of_pancreas	uterus	lung
chr8	52558395	rs12548130	C	A	INT	Intelligent	large_intestine	small_intestine	heart_right_ventricle	brain	eye
chr4	183497776	rs9996737	T	G	INT	Intelligent	retina	eye	placenta	lung	right_lung
chr18	50192001	rs7242423	G	A	MDD	Major depressive disorder	left_lung	lung	right_lung	forelimb_muscle	hindlimb_muscle
chr17	45732605	rs11870935	G	A	MS	Multiple sclerosis	small_intestine	transverse_colon	large_intestine	body_of_pancreas	pancreas
chr6	30697447	rs3094127	A	G	MS	Multiple sclerosis	small_intestine	transverse_colon	large_intestine	body_of_pancreas	Peyer's_patch
chr6	32398202	rs9268589	G	A	MS	Multiple sclerosis	small_intestine	large_intestine	transverse_colon	Peyer's_patch	body_of_pancreas
chr6	30797083	rs9368644	C	T	MS	Multiple sclerosis	transverse_colon	small_intestine	large_intestine	body_of_pancreas	Peyer's_patch
chr1	98524960	rs1702292	A	G	SCZ	Schizophrenia	placenta	heart_right_ventricle	brain	retina	eye
chr3	126792822	rs4375997	A	G	SCZ	Schizophrenia	placenta	body_of_pancreas	breast_epithelium	pancreas	transverse_colon
chr17	46037607	rs12602494	G	A	T1D	Type 1 diabetes	large_intestine	small_intestine	transverse_colon	Peyer's_patch	body_of_pancreas
chr15	82054987	rs12916962	C	T	T1D	Type 1 diabetes	small_intestine	large_intestine	transverse_colon	Peyer's_patch	body_of_pancreas
chr16	11242858	rs794426	C	A	T1D	Type 1 diabetes	large_intestine	small_intestine	transverse_colon	Peyer's_patch	body_of_pancreas
chr7	28189411	rs1635852	T	C	T2D	Type 2 diabetes	small_intestine	transverse_colon	large_intestine	body_of_pancreas	Peyer's_patch
chr5	66312570	rs628763	T	G	T2D	Type 2 diabetes	right_kidney	renal_pelvis	right_renal_pelvis	left_kidney	renal_cortex_interstitium
chr4	96147860	rs6850673	T	C	T2D	Type 2 diabetes	pancreas	body_of_pancreas	lung	eye	heart_left_ventricle
chr6	32491056	rs111503621	A	G	UC	Ulcerative colitis	small_intestine	large_intestine	transverse_colon	eye	Peyer's_patch
chr6	32485583	rs113045321	T	C	UC	Ulcerative colitis	transverse_colon	body_of_pancreas	small_intestine	pancreas	large_intestine
chr6	31040306	rs117788300	T	A	UC	Ulcerative colitis	transverse_colon	body_of_pancreas	small_intestine	large_intestine	Peyer's_patch
chr6	32536497	rs147611303	G	A	UC	Ulcerative colitis	psoas_muscle	adrenal_gland	pancreas	placenta	left_kidney
chr6	32561766	rs28366328	A	G	UC	Ulcerative colitis	heart_right_ventricle	small_intestine	large_intestine	heart	heart_left_ventricle
chr6	32600003	rs3104376	T	C	UC	Ulcerative colitis	small_intestine	large_intestine	transverse_colon	heart_right_ventricle	heart_left_ventricle
chr6	32521062	rs3828833	T	C	UC	Ulcerative colitis	small_intestine	large_intestine	transverse_colon	body_of_pancreas	Peyer's_patch
chr6	31320930	rs77616974	C	G	UC	Ulcerative colitis	Peyer's_patch	small_intestine	transverse_colon	large_intestine	body_of_pancreas
chr6	32623073	rs79692442	T	C	UC	Ulcerative colitis	small_intestine	large_intestine	transverse_colon	pancreas	placenta
chr6	32566533	rs9270670	A	G	UC	Ulcerative colitis	transverse_colon	small_intestine	large_intestine	pancreas	spleen
chr6	32640149	rs9274848	C	G	UC	Ulcerative colitis	small_intestine	large_intestine	transverse_colon	body_of_pancreas	Peyer's_patch
chr6	32640157	rs9274849	A	T	UC	Ulcerative colitis	transverse_colon	small_intestine	large_intestine	body_of_pancreas	Peyer's_patch

* The row represents variant, the column represents variant's detailed information. Here, we rank the varaint's tissue-specificity (relevant) base on their median SAD score.

Supplementary Table S2. Validation results of *in silico* saturated mutagenesis analysis.

Variant	Position	Profile_index	Profile_name	Max_loss	Max_gain
chr1_20171860_G_A_G	-99	2133	NFE2-human^cell_line^K562^ENCFF813HVG	0	0.003172636
chr1_20171860_G_A_G	-98	2133	NFE2-human^cell_line^K562^ENCFF813HVG	0.003272504	0.001584351
chr1_20171860_G_A_G	-97	2133	NFE2-human^cell_line^K562^ENCFF813HVG	0.006067693	0.002589554
chr1_20171860_G_A_G	-96	2133	NFE2-human^cell_line^K562^ENCFF813HVG	0.007717907	0.002443671
chr1_20171860_G_A_G	-95	2133	NFE2-human^cell_line^K562^ENCFF813HVG	0.007056445	0.000408113
chr1_20171860_G_A_G	-94	2133	NFE2-human^cell_line^K562^ENCFF813HVG	0.005356461	0.003973126
chr1_20171860_G_A_G	-93	2133	NFE2-human^cell_line^K562^ENCFF813HVG	0.003640562	0.001388997
chr1_20171860_G_A_G	-92	2133	NFE2-human^cell_line^K562^ENCFF813HVG	0.010337681	0.001145244
chr1_20171860_G_A_G	-91	2133	NFE2-human^cell_line^K562^ENCFF813HVG	0.007362872	0.000769377
chr1_20171860_G_A_G	-90	2133	NFE2-human^cell_line^K562^ENCFF813HVG	0.002442926	0
chr1_20171860_G_A_G	-89	2133	NFE2-human^cell_line^K562^ENCFF813HVG	0.004951388	0
chr1_20171860_G_A_G	-88	2133	NFE2-human^cell_line^K562^ENCFF813HVG	0.009115934	0
chr1_20171860_G_A_G	-87	2133	NFE2-human^cell_line^K562^ENCFF813HVG	0.005671591	0.005043507
chr1_20171860_G_A_G	-86	2133	NFE2-human^cell_line^K562^ENCFF813HVG	0.008876652	0
chr1_20171860_G_A_G	-85	2133	NFE2-human^cell_line^K562^ENCFF813HVG	0.005711883	0.010453641
chr1_20171860_G_A_G	-84	2133	NFE2-human^cell_line^K562^ENCFF813HVG	0	0.008821547
chr1_20171860_G_A_G	-83	2133	NFE2-human^cell_line^K562^ENCFF813HVG	0.012169719	0
chr1_20171860_G_A_G	-82	2133	NFE2-human^cell_line^K562^ENCFF813HVG	0.011159003	0.007134408
chr1_20171860_G_A_G	-81	2133	NFE2-human^cell_line^K562^ENCFF813HVG	0.002095282	0.00581044
chr1_20171860_G_A_G	-80	2133	NFE2-human^cell_line^K562^ENCFF813HVG	0.004354715	0.001091093
chr1_20171860_G_A_G	-79	2133	NFE2-human^cell_line^K562^ENCFF813HVG	0	0.010363102
chr1_20171860_G_A_G	-78	2133	NFE2-human^cell_line^K562^ENCFF813HVG	0.010076791	0
chr1_20171860_G_A_G	-77	2133	NFE2-human^cell_line^K562^ENCFF813HVG	0.001867563	0.013948023
chr1_20171860_G_A_G	-76	2133	NFE2-human^cell_line^K562^ENCFF813HVG	0.006406486	0.004994571
chr1_20171860_G_A_G	-75	2133	NFE2-human^cell_line^K562^ENCFF813HVG	0.002293587	0.01168859
chr1_20171860_G_A_G	-74	2133	NFE2-human^cell_line^K562^ENCFF813HVG	0.007235765	0.007377863
chr1_20171860_G_A_G	-73	2133	NFE2-human^cell_line^K562^ENCFF813HVG	0.000813425	0.00962016
chr1_20171860_G_A_G	-72	2133	NFE2-human^cell_line^K562^ENCFF813HVG	0.005597472	0.007576257
chr1_20171860_G_A_G	-71	2133	NFE2-human^cell_line^K562^ENCFF813HVG	0.007889509	0.00384587
chr1_20171860_G_A_G	-70	2133	NFE2-human^cell_line^K562^ENCFF813HVG	0	0.00839442
chr1_20171860_G_A_G	-69	2133	NFE2-human^cell_line^K562^ENCFF813HVG	0.007128239	0
chr1_20171860_G_A_G	-68	2133	NFE2-human^cell_line^K562^ENCFF813HVG	0	0.008929491
chr1_20171860_G_A_G	-67	2133	NFE2-human^cell_line^K562^ENCFF813HVG	0.004267603	0.011256009
chr1_20171860_G_A_G	-66	2133	NFE2-human^cell_line^K562^ENCFF813HVG	0	0.012710363
chr1_20171860_G_A_G	-65	2133	NFE2-human^cell_line^K562^ENCFF813HVG	0.008171082	0
chr1_20171860_G_A_G	-64	2133	NFE2-human^cell_line^K562^ENCFF813HVG	0.001827061	2.47E-06
chr1_20171860_G_A_G	-63	2133	NFE2-human^cell_line^K562^ENCFF813HVG	0.000455022	0.002650291
chr1_20171860_G_A_G	-62	2133	NFE2-human^cell_line^K562^ENCFF813HVG	0.009571463	0.000911266
chr1_20171860_G_A_G	-61	2133	NFE2-human^cell_line^K562^ENCFF813HVG	0	0.013278961
chr1_20171860_G_A_G	-60	2133	NFE2-human^cell_line^K562^ENCFF813HVG	0.003330886	0.006976992
chr1_20171860_G_A_G	-59	2133	NFE2-human^cell_line^K562^ENCFF813HVG	0	0.010366291
chr1_20171860_G_A_G	-58	2133	NFE2-human^cell_line^K562^ENCFF813HVG	0	0.01658842
chr1_20171860_G_A_G	-57	2133	NFE2-human^cell_line^K562^ENCFF813HVG	0	0.004747361
chr1_20171860_G_A_G	-56	2133	NFE2-human^cell_line^K562^ENCFF813HVG	0	0.014903158
chr1_20171860_G_A_G	-55	2133	NFE2-human^cell_line^K562^ENCFF813HVG	0.005683392	0
chr1_20171860_G_A_G	-54	2133	NFE2-human^cell_line^K562^ENCFF813HVG	0	0.023321062
chr1_20171860_G_A_G	-53	2133	NFE2-human^cell_line^K562^ENCFF813HVG	0.008369207	0
chr1_20171860_G_A_G	-52	2133	NFE2-human^cell_line^K562^ENCFF813HVG	0.008464009	0
chr1_20171860_G_A_G	-51	2133	NFE2-human^cell_line^K562^ENCFF813HVG	0.007469416	0.001962066
chr1_20171860_G_A_G	-50	2133	NFE2-human^cell_line^K562^ENCFF813HVG	0.016186178	0
chr1_20171860_G_A_G	-49	2133	NFE2-human^cell_line^K562^ENCFF813HVG	0.001444131	0.006547689
chr1_20171860_G_A_G	-48	2133	NFE2-human^cell_line^K562^ENCFF813HVG	0.009613514	0
chr1_20171860_G_A_G	-47	2133	NFE2-human^cell_line^K562^ENCFF813HVG	0.005211174	0.006744295
chr1_20171860_G_A_G	-46	2133	NFE2-human^cell_line^K562^ENCFF813HVG	0.019141138	0
chr1_20171860_G_A_G	-45	2133	NFE2-human^cell_line^K562^ENCFF813HVG	0.011109889	0.031852901
chr1_20171860_G_A_G	-44	2133	NFE2-human^cell_line^K562^ENCFF813HVG	0.006236523	0
chr1_20171860_G_A_G	-43	2133	NFE2-human^cell_line^K562^ENCFF813HVG	0.014526665	0.003247708
chr1_20171860_G_A_G	-42	2133	NFE2-human^cell_line^K562^ENCFF813HVG	0	0.089532793
chr1_20171860_G_A_G	-41	2133	NFE2-human^cell_line^K562^ENCFF813HVG	0.010302752	0.008870363
chr1_20171860_G_A_G	-40	2133	NFE2-human^cell_line^K562^ENCFF813HVG	0.007878155	0.001136333
chr1_20171860_G_A_G	-39	2133	NFE2-human^cell_line^K562^ENCFF813HVG	0	0.008383572
chr1_20171860_G_A_G	-38	2133	NFE2-human^cell_line^K562^ENCFF813HVG	0.004183292	0
chr1_20171860_G_A_G	-37	2133	NFE2-human^cell_line^K562^ENCFF813HVG	0.005798876	0
chr1_20171860_G_A_G	-36	2133	NFE2-human^cell_line^K562^ENCFF813HVG	0.003269523	0.006765515
chr1_20171860_G_A_G	-35	2133	NFE2-human^cell_line^K562^ENCFF813HVG	0	0.007557005
chr1_20171860_G_A_G	-34	2133	NFE2-human^cell_line^K562^ENCFF813HVG	0	0.0110991
chr1_20171860_G_A_G	-33	2133	NFE2-human^cell_line^K562^ENCFF813HVG	0	0.018911868
chr1_20171860_G_A_G	-32	2133	NFE2-human^cell_line^K562^ENCFF813HVG	0.000588536	0.009248108
chr1_20171860_G_A_G	-31	2133	NFE2-human^cell_line^K562^ENCFF813HVG	0	0.007688791
chr1_20171860_G_A_G	-30	2133	NFE2-human^cell_line^K562^ENCFF813HVG	0.00371787	0.007567197
chr1_20171860_G_A_G	-29	2133	NFE2-human^cell_line^K562^ENCFF813HVG	0.009836316	0
chr1_20171860_G_A_G	-28	2133	NFE2-human^cell_line^K562^ENCFF813HVG	0.01248014	0
chr1_20171860_G_A_G	-27	2133	NFE2-human^cell_line^K562^ENCFF813HVG	0.000524074	0.009231329
chr1_20171860_G_A_G	-26	2133	NFE2-human^cell_line^K562^ENCFF813HVG	0.009717971	0
chr1_20171860_G_A_G	-25	2133	NFE2-human^cell_line^K562^ENCFF813HVG	0	0.005814582
chr1_20171860_G_A_G	-24	2133	NFE2-human^cell_line^K562^ENCFF813HVG	0.007115155	0.008321524
chr1_20171860_G_A_G	-23	2133	NFE2-human^cell_line^K562^ENCFF813HVG	0.00385794	0.002020299
chr1_20171860_G_A_G	-22	2133	NFE2-human^cell_line^K562^ENCFF813HVG	0.004270881	0.002897054
chr1_20171860_G_A_G	-21	2133	NFE2-human^cell_line^K562^ENCFF813HVG	0.013515532	0
chr1_20171860_G_A_G	-20	2133	NFE2-human^cell_line^K562^ENCFF813HVG	0.008418024	0
chr1_20171860_G_A_G	-19	2133	NFE2-human^cell_line^K562^ENCFF813HVG	0.021409631	0
chr1_20171860_G_A_G	-18	2133	NFE2-human^cell_line^K562^ENCFF813HVG	0.017038971	0
chr1_20171860_G_A_G	-17	2133	NFE2-human^cell_line^K562^ENCFF813HVG	0.004301459	0.006748945

chr1_20171860_G_A_G	-16	2133	NFE2-human^cell_line^K562^ENCFF813HVG	0.007150829	0.009597689
chr1_20171860_G_A_G	-15	2133	NFE2-human^cell_line^K562^ENCFF813HVG	0.00512749	0.000974029
chr1_20171860_G_A_G	-14	2133	NFE2-human^cell_line^K562^ENCFF813HVG	0.008300245	0
chr1_20171860_G_A_G	-13	2133	NFE2-human^cell_line^K562^ENCFF813HVG	0.022021443	0
chr1_20171860_G_A_G	-12	2133	NFE2-human^cell_line^K562^ENCFF813HVG	0.013343245	0.007116407
chr1_20171860_G_A_G	-11	2133	NFE2-human^cell_line^K562^ENCFF813HVG	0.017328084	0.005627453
chr1_20171860_G_A_G	-10	2133	NFE2-human^cell_line^K562^ENCFF813HVG	0.010081172	0.006410748
chr1_20171860_G_A_G	-9	2133	NFE2-human^cell_line^K562^ENCFF813HVG	0	0.008887857
chr1_20171860_G_A_G	-8	2133	NFE2-human^cell_line^K562^ENCFF813HVG	0.025507301	0
chr1_20171860_G_A_G	-7	2133	NFE2-human^cell_line^K562^ENCFF813HVG	0.016697079	0.000716805
chr1_20171860_G_A_G	-6	2133	NFE2-human^cell_line^K562^ENCFF813HVG	0.018346876	0
chr1_20171860_G_A_G	-5	2133	NFE2-human^cell_line^K562^ENCFF813HVG	0.014081627	0
chr1_20171860_G_A_G	-4	2133	NFE2-human^cell_line^K562^ENCFF813HVG	0.017083913	0
chr1_20171860_G_A_G	-3	2133	NFE2-human^cell_line^K562^ENCFF813HVG	0.015231103	0.02728197
chr1_20171860_G_A_G	-2	2133	NFE2-human^cell_line^K562^ENCFF813HVG	0.009120762	0
chr1_20171860_G_A_G	-1	2133	NFE2-human^cell_line^K562^ENCFF813HVG	0.017774016	0
chr1_20171860_G_A_G	**0**	**2133**	**NFE2-human^cell_line^K562^ENCFF813HVG**	**0.022096515**	**0.124233931**
chr1_20171860_G_A_G	1	2133	NFE2-human^cell_line^K562^ENCFF813HVG	0.013015032	0.027857721
chr1_20171860_G_A_G	2	2133	NFE2-human^cell_line^K562^ENCFF813HVG	0.011811137	0
chr1_20171860_G_A_G	3	2133	NFE2-human^cell_line^K562^ENCFF813HVG	0.00763607	0.001751989
chr1_20171860_G_A_G	4	2133	NFE2-human^cell_line^K562^ENCFF813HVG	0.005259216	0.015052766
chr1_20171860_G_A_G	5	2133	NFE2-human^cell_line^K562^ENCFF813HVG	0.022220165	0.003688544
chr1_20171860_G_A_G	6	2133	NFE2-human^cell_line^K562^ENCFF813HVG	0	0.00486356
chr1_20171860_G_A_G	7	2133	NFE2-human^cell_line^K562^ENCFF813HVG	4.67E-05	0.011307776
chr1_20171860_G_A_G	8	2133	NFE2-human^cell_line^K562^ENCFF813HVG	0.008829355	0.006852448
chr1_20171860_G_A_G	9	2133	NFE2-human^cell_line^K562^ENCFF813HVG	0.000682086	0.014089435
chr1_20171860_G_A_G	10	2133	NFE2-human^cell_line^K562^ENCFF813HVG	0.000346392	0.013669461
chr1_20171860_G_A_G	11	2133	NFE2-human^cell_line^K562^ENCFF813HVG	0.005458176	0.011087179
chr1_20171860_G_A_G	12	2133	NFE2-human^cell_line^K562^ENCFF813HVG	0	0.005774826
chr1_20171860_G_A_G	13	2133	NFE2-human^cell_line^K562^ENCFF813HVG	0	0.021818995
chr1_20171860_G_A_G	14	2133	NFE2-human^cell_line^K562^ENCFF813HVG	0	0.012179881
chr1_20171860_G_A_G	15	2133	NFE2-human^cell_line^K562^ENCFF813HVG	0.011005849	0.020849109
chr1_20171860_G_A_G	16	2133	NFE2-human^cell_line^K562^ENCFF813HVG	0	0.036844492
chr1_20171860_G_A_G	17	2133	NFE2-human^cell_line^K562^ENCFF813HVG	0.002641171	0.004813671
chr1_20171860_G_A_G	18	2133	NFE2-human^cell_line^K562^ENCFF813HVG	0.011648834	0
chr1_20171860_G_A_G	19	2133	NFE2-human^cell_line^K562^ENCFF813HVG	0.011290908	0.007537782
chr1_20171860_G_A_G	20	2133	NFE2-human^cell_line^K562^ENCFF813HVG	0.007849723	0.009781986
chr1_20171860_G_A_G	21	2133	NFE2-human^cell_line^K562^ENCFF813HVG	0.004314363	0.053865671
chr1_20171860_G_A_G	22	2133	NFE2-human^cell_line^K562^ENCFF813HVG	0	0.006688356
chr1_20171860_G_A_G	23	2133	NFE2-human^cell_line^K562^ENCFF813HVG	0.016140193	0
chr1_20171860_G_A_G	24	2133	NFE2-human^cell_line^K562^ENCFF813HVG	0.004184902	0.006043583
chr1_20171860_G_A_G	25	2133	NFE2-human^cell_line^K562^ENCFF813HVG	0.001027554	0.000553936
chr1_20171860_G_A_G	26	2133	NFE2-human^cell_line^K562^ENCFF813HVG	0.002525896	0.029055476
chr1_20171860_G_A_G	27	2133	NFE2-human^cell_line^K562^ENCFF813HVG	0.00872758	0.003651321
chr1_20171860_G_A_G	28	2133	NFE2-human^cell_line^K562^ENCFF813HVG	0.000560969	0.020148337
chr1_20171860_G_A_G	29	2133	NFE2-human^cell_line^K562^ENCFF813HVG	0.005661607	0
chr1_20171860_G_A_G	30	2133	NFE2-human^cell_line^K562^ENCFF813HVG	0.015207022	0
chr1_20171860_G_A_G	31	2133	NFE2-human^cell_line^K562^ENCFF813HVG	0.012193352	0
chr1_20171860_G_A_G	32	2133	NFE2-human^cell_line^K562^ENCFF813HVG	0.006076217	0
chr1_20171860_G_A_G	33	2133	NFE2-human^cell_line^K562^ENCFF813HVG	0.01527676	0.003105551
chr1_20171860_G_A_G	34	2133	NFE2-human^cell_line^K562^ENCFF813HVG	0.004891425	0.004408717
chr1_20171860_G_A_G	35	2133	NFE2-human^cell_line^K562^ENCFF813HVG	0.004170477	0.016733736
chr1_20171860_G_A_G	36	2133	NFE2-human^cell_line^K562^ENCFF813HVG	0.00389418	0.01041761
chr1_20171860_G_A_G	37	2133	NFE2-human^cell_line^K562^ENCFF813HVG	0	0.039025754
chr1_20171860_G_A_G	38	2133	NFE2-human^cell_line^K562^ENCFF813HVG	0	0.003353983
chr1_20171860_G_A_G	39	2133	NFE2-human^cell_line^K562^ENCFF813HVG	0.006331205	0.003009319
chr1_20171860_G_A_G	40	2133	NFE2-human^cell_line^K562^ENCFF813HVG	0.002587408	0.005446911
chr1_20171860_G_A_G	41	2133	NFE2-human^cell_line^K562^ENCFF813HVG	0.006335199	0.002192885
chr1_20171860_G_A_G	42	2133	NFE2-human^cell_line^K562^ENCFF813HVG	0.004137367	0
chr1_20171860_G_A_G	43	2133	NFE2-human^cell_line^K562^ENCFF813HVG	0.007500201	0
chr1_20171860_G_A_G	44	2133	NFE2-human^cell_line^K562^ENCFF813HVG	0.009114742	0.000462413
chr1_20171860_G_A_G	45	2133	NFE2-human^cell_line^K562^ENCFF813HVG	0.001148432	0.004296213
chr1_20171860_G_A_G	46	2133	NFE2-human^cell_line^K562^ENCFF813HVG	0.004008234	0.003029943
chr1_20171860_G_A_G	47	2133	NFE2-human^cell_line^K562^ENCFF813HVG	0.012299329	0
chr1_20171860_G_A_G	48	2133	NFE2-human^cell_line^K562^ENCFF813HVG	0	0.004695058
chr1_20171860_G_A_G	49	2133	NFE2-human^cell_line^K562^ENCFF813HVG	0	0.007099539
chr1_20171860_G_A_G	50	2133	NFE2-human^cell_line^K562^ENCFF813HVG	0.000506639	0.00232172
chr1_20171860_G_A_G	51	2133	NFE2-human^cell_line^K562^ENCFF813HVG	0	0.002736807
chr1_20171860_G_A_G	52	2133	NFE2-human^cell_line^K562^ENCFF813HVG	0	0.009736806
chr1_20171860_G_A_G	53	2133	NFE2-human^cell_line^K562^ENCFF813HVG	0.007106721	0
chr1_20171860_G_A_G	54	2133	NFE2-human^cell_line^K562^ENCFF813HVG	0	0.007139236
chr1_20171860_G_A_G	55	2133	NFE2-human^cell_line^K562^ENCFF813HVG	0.006876975	0.011686981
chr1_20171860_G_A_G	56	2133	NFE2-human^cell_line^K562^ENCFF813HVG	0.001779228	0.004655302
chr1_20171860_G_A_G	57	2133	NFE2-human^cell_line^K562^ENCFF813HVG	0	0.018248498
chr1_20171860_G_A_G	58	2133	NFE2-human^cell_line^K562^ENCFF813HVG	0.003696024	0.005951256
chr1_20171860_G_A_G	59	2133	NFE2-human^cell_line^K562^ENCFF813HVG	0.000692308	0.006425858
chr1_20171860_G_A_G	60	2133	NFE2-human^cell_line^K562^ENCFF813HVG	0.005665004	0.000729531
chr1_20171860_G_A_G	61	2133	NFE2-human^cell_line^K562^ENCFF813HVG	0.001413435	0.01060459
chr1_20171860_G_A_G	62	2133	NFE2-human^cell_line^K562^ENCFF813HVG	0.005014777	3.05E-05
chr1_20171860_G_A_G	63	2133	NFE2-human^cell_line^K562^ENCFF813HVG	0	0.009977847
chr1_20171860_G_A_G	64	2133	NFE2-human^cell_line^K562^ENCFF813HVG	0	0.005658776
chr1_20171860_G_A_G	65	2133	NFE2-human^cell_line^K562^ENCFF813HVG	0.011050671	0
chr1_20171860_G_A_G	66	2133	NFE2-human^cell_line^K562^ENCFF813HVG	0.00427717	0.003685325
chr1_20171860_G_A_G	67	2133	NFE2-human^cell_line^K562^ENCFF813HVG	0.00914064	0
chr1_20171860_G_A_G	68	2133	NFE2-human^cell_line^K562^ENCFF813HVG	0.002364755	0.001823485
chr1_20171860_G_A_G	69	2133	NFE2-human^cell_line^K562^ENCFF813HVG	0.007938117	0

chr1_20171860_G_A_G	70	2133	NFE2-human^cell_line^K562^ENCFF813HVG	0.001811951	0
chr1_20171860_G_A_G	71	2133	NFE2-human^cell_line^K562^ENCFF813HVG	0	0.003375739
chr1_20171860_G_A_G	72	2133	NFE2-human^cell_line^K562^ENCFF813HVG	0.008740723	0
chr1_20171860_G_A_G	73	2133	NFE2-human^cell_line^K562^ENCFF813HVG	0.001265883	0.002088428
chr1_20171860_G_A_G	74	2133	NFE2-human^cell_line^K562^ENCFF813HVG	0.007913232	0
chr1_20171860_G_A_G	75	2133	NFE2-human^cell_line^K562^ENCFF813HVG	0.001359344	0.000358015
chr1_20171860_G_A_G	76	2133	NFE2-human^cell_line^K562^ENCFF813HVG	0.00539124	0
chr1_20171860_G_A_G	77	2133	NFE2-human^cell_line^K562^ENCFF813HVG	0	0.006143689
chr1_20171860_G_A_G	78	2133	NFE2-human^cell_line^K562^ENCFF813HVG	0.009090573	5.45E-06
chr1_20171860_G_A_G	79	2133	NFE2-human^cell_line^K562^ENCFF813HVG	0.004803538	0
chr1_20171860_G_A_G	80	2133	NFE2-human^cell_line^K562^ENCFF813HVG	0.004909337	0
chr1_20171860_G_A_G	81	2133	NFE2-human^cell_line^K562^ENCFF813HVG	0.008160472	0
chr1_20171860_G_A_G	82	2133	NFE2-human^cell_line^K562^ENCFF813HVG	0.008188725	0
chr1_20171860_G_A_G	83	2133	NFE2-human^cell_line^K562^ENCFF813HVG	0.008417666	0
chr1_20171860_G_A_G	84	2133	NFE2-human^cell_line^K562^ENCFF813HVG	0.001408249	0.009026557
chr1_20171860_G_A_G	85	2133	NFE2-human^cell_line^K562^ENCFF813HVG	0	0.008282304
chr1_20171860_G_A_G	86	2133	NFE2-human^cell_line^K562^ENCFF813HVG	0.003614545	0
chr1_20171860_G_A_G	87	2133	NFE2-human^cell_line^K562^ENCFF813HVG	0.001680672	0.002118558
chr1_20171860_G_A_G	88	2133	NFE2-human^cell_line^K562^ENCFF813HVG	0.003020257	0.00669679
chr1_20171860_G_A_G	89	2133	NFE2-human^cell_line^K562^ENCFF813HVG	0.001922011	0.004230946
chr1_20171860_G_A_G	90	2133	NFE2-human^cell_line^K562^ENCFF813HVG	0.003546268	0.008470476
chr1_20171860_G_A_G	91	2133	NFE2-human^cell_line^K562^ENCFF813HVG	0.007633954	0.000881284
chr1_20171860_G_A_G	92	2133	NFE2-human^cell_line^K562^ENCFF813HVG	0.008104295	0
chr1_20171860_G_A_G	93	2133	NFE2-human^cell_line^K562^ENCFF813HVG	0.004988819	0.001271695
chr1_20171860_G_A_G	94	2133	NFE2-human^cell_line^K562^ENCFF813HVG	0.002324909	0.002494961
chr1_20171860_G_A_G	95	2133	NFE2-human^cell_line^K562^ENCFF813HVG	0.000267893	0.002176732
chr1_20171860_G_A_G	96	2133	NFE2-human^cell_line^K562^ENCFF813HVG	0.002547413	0.003691375
chr1_20171860_G_A_G	97	2133	NFE2-human^cell_line^K562^ENCFF813HVG	0	0.006158859
chr1_20171860_G_A_G	98	2133	NFE2-human^cell_line^K562^ENCFF813HVG	0.003009766	0.001421601
chr1_20171860_G_A_G	99	2133	NFE2-human^cell_line^K562^ENCFF813HVG	0.002519369	0.002116561
chr1_20171860_G_A_G	100	2133	NFE2-human^cell_line^K562^ENCFF813HVG	0.009542614	0
chr1_20171860_G_A_A	-99	2133	NFE2-human^cell_line^K562^ENCFF813HVG	0	0.002502382
chr1_20171860_G_A_A	-98	2133	NFE2-human^cell_line^K562^ENCFF813HVG	0.003712863	0.000988692
chr1_20171860_G_A_A	-97	2133	NFE2-human^cell_line^K562^ENCFF813HVG	0.00729847	0.001719862
chr1_20171860_G_A_A	-96	2133	NFE2-human^cell_line^K562^ENCFF813HVG	0.009075582	0.002231419
chr1_20171860_G_A_A	-95	2133	NFE2-human^cell_line^K562^ENCFF813HVG	0.007582188	0.000482798
chr1_20171860_G_A_A	-94	2133	NFE2-human^cell_line^K562^ENCFF813HVG	0.006449878	0.003352761
chr1_20171860_G_A_A	-93	2133	NFE2-human^cell_line^K562^ENCFF813HVG	0.003773063	0.000903249
chr1_20171860_G_A_A	-92	2133	NFE2-human^cell_line^K562^ENCFF813HVG	0.010875344	0.001107752
chr1_20171860_G_A_A	-91	2133	NFE2-human^cell_line^K562^ENCFF813HVG	0.008511335	0.000422031
chr1_20171860_G_A_A	-90	2133	NFE2-human^cell_line^K562^ENCFF813HVG	0.00360024	0
chr1_20171860_G_A_A	-89	2133	NFE2-human^cell_line^K562^ENCFF813HVG	0.006681204	0
chr1_20171860_G_A_A	-88	2133	NFE2-human^cell_line^K562^ENCFF813HVG	0.010552377	0
chr1_20171860_G_A_A	-87	2133	NFE2-human^cell_line^K562^ENCFF813HVG	0.005913585	0.004061401
chr1_20171860_G_A_A	-86	2133	NFE2-human^cell_line^K562^ENCFF813HVG	0.010654062	0
chr1_20171860_G_A_A	-85	2133	NFE2-human^cell_line^K562^ENCFF813HVG	0.006211519	0.00947535
chr1_20171860_G_A_A	-84	2133	NFE2-human^cell_line^K562^ENCFF813HVG	0	0.008428246
chr1_20171860_G_A_A	-83	2133	NFE2-human^cell_line^K562^ENCFF813HVG	0.013735086	0
chr1_20171860_G_A_A	-82	2133	NFE2-human^cell_line^K562^ENCFF813HVG	0.013869822	0.004497617
chr1_20171860_G_A_A	-81	2133	NFE2-human^cell_line^K562^ENCFF813HVG	0.003557771	0.005373776
chr1_20171860_G_A_A	-80	2133	NFE2-human^cell_line^K562^ENCFF813HVG	0.006170183	0
chr1_20171860_G_A_A	-79	2133	NFE2-human^cell_line^K562^ENCFF813HVG	0	0.009816974
chr1_20171860_G_A_A	-78	2133	NFE2-human^cell_line^K562^ENCFF813HVG	0.013744146	0
chr1_20171860_G_A_A	-77	2133	NFE2-human^cell_line^K562^ENCFF813HVG	0.00320673	0.012774318
chr1_20171860_G_A_A	-76	2133	NFE2-human^cell_line^K562^ENCFF813HVG	0.00898087	0.003198147
chr1_20171860_G_A_A	-75	2133	NFE2-human^cell_line^K562^ENCFF813HVG	0.001928687	0.013340205
chr1_20171860_G_A_A	-74	2133	NFE2-human^cell_line^K562^ENCFF813HVG	0.00829348	0.008027196
chr1_20171860_G_A_A	-73	2133	NFE2-human^cell_line^K562^ENCFF813HVG	0.004444033	0.01023069
chr1_20171860_G_A_A	-72	2133	NFE2-human^cell_line^K562^ENCFF813HVG	0.00615257	0.008025587
chr1_20171860_G_A_A	-71	2133	NFE2-human^cell_line^K562^ENCFF813HVG	0.011544853	0.00278163
chr1_20171860_G_A_A	-70	2133	NFE2-human^cell_line^K562^ENCFF813HVG	0.004296064	0.007561088
chr1_20171860_G_A_A	-69	2133	NFE2-human^cell_line^K562^ENCFF813HVG	0.011892885	0
chr1_20171860_G_A_A	-68	2133	NFE2-human^cell_line^K562^ENCFF813HVG	0	0.007863164
chr1_20171860_G_A_A	-67	2133	NFE2-human^cell_line^K562^ENCFF813HVG	0.005340517	0.018013895
chr1_20171860_G_A_A	-66	2133	NFE2-human^cell_line^K562^ENCFF813HVG	0	0.011793226
chr1_20171860_G_A_A	-65	2133	NFE2-human^cell_line^K562^ENCFF813HVG	0.0138762	0
chr1_20171860_G_A_A	-64	2133	NFE2-human^cell_line^K562^ENCFF813HVG	0.003033876	0
chr1_20171860_G_A_A	-63	2133	NFE2-human^cell_line^K562^ENCFF813HVG	0.00246501	0.002251536
chr1_20171860_G_A_A	-62	2133	NFE2-human^cell_line^K562^ENCFF813HVG	0.014931291	0
chr1_20171860_G_A_A	-61	2133	NFE2-human^cell_line^K562^ENCFF813HVG	0	0.01267755
chr1_20171860_G_A_A	-60	2133	NFE2-human^cell_line^K562^ENCFF813HVG	0.008371413	0.005149633
chr1_20171860_G_A_A	-59	2133	NFE2-human^cell_line^K562^ENCFF813HVG	0	0.015227199
chr1_20171860_G_A_A	-58	2133	NFE2-human^cell_line^K562^ENCFF813HVG	0	0.019879729
chr1_20171860_G_A_A	-57	2133	NFE2-human^cell_line^K562^ENCFF813HVG	0	0.005656779
chr1_20171860_G_A_A	-56	2133	NFE2-human^cell_line^K562^ENCFF813HVG	0	0.024051309
chr1_20171860_G_A_A	-55	2133	NFE2-human^cell_line^K562^ENCFF813HVG	0.010256857	0
chr1_20171860_G_A_A	-54	2133	NFE2-human^cell_line^K562^ENCFF813HVG	0	0.031713247
chr1_20171860_G_A_A	-53	2133	NFE2-human^cell_line^K562^ENCFF813HVG	0.015397161	0
chr1_20171860_G_A_A	-52	2133	NFE2-human^cell_line^K562^ENCFF813HVG	0.007959008	0
chr1_20171860_G_A_A	-51	2133	NFE2-human^cell_line^K562^ENCFF813HVG	0.012596309	0
chr1_20171860_G_A_A	-50	2133	NFE2-human^cell_line^K562^ENCFF813HVG	0.024604052	0
chr1_20171860_G_A_A	-49	2133	NFE2-human^cell_line^K562^ENCFF813HVG	0.006868631	0.01348877
chr1_20171860_G_A_A	-48	2133	NFE2-human^cell_line^K562^ENCFF813HVG	0.017032623	0
chr1_20171860_G_A_A	-47	2133	NFE2-human^cell_line^K562^ENCFF813HVG	0.01102984	0.010042906
chr1_20171860_G_A_A	-46	2133	NFE2-human^cell_line^K562^ENCFF813HVG	0.029197395	0

chr1_20171860_G_A_A	-45	2133	NFE2-human^cell_line^K562^ENCFF813HVG	0.015107185	0.038777739
chr1_20171860_G_A_A	-44	2133	NFE2-human^cell_line^K562^ENCFF813HVG	0.00921005	0
chr1_20171860_G_A_A	-43	2133	NFE2-human^cell_line^K562^ENCFF813HVG	0.021237612	0.007439733
chr1_20171860_G_A_A	-42	2133	NFE2-human^cell_line^K562^ENCFF813HVG	0	0.089007467
chr1_20171860_G_A_A	-41	2133	NFE2-human^cell_line^K562^ENCFF813HVG	0.016194552	0.008966446
chr1_20171860_G_A_A	-40	2133	NFE2-human^cell_line^K562^ENCFF813HVG	0.013135403	0.001888722
chr1_20171860_G_A_A	-39	2133	NFE2-human^cell_line^K562^ENCFF813HVG	0	0.01232788
chr1_20171860_G_A_A	-38	2133	NFE2-human^cell_line^K562^ENCFF813HVG	0.002941579	0
chr1_20171860_G_A_A	-37	2133	NFE2-human^cell_line^K562^ENCFF813HVG	0.010393143	0.003037274
chr1_20171860_G_A_A	-36	2133	NFE2-human^cell_line^K562^ENCFF813HVG	0.005133629	0.010268778
chr1_20171860_G_A_A	-35	2133	NFE2-human^cell_line^K562^ENCFF813HVG	0	0.009384573
chr1_20171860_G_A_A	-34	2133	NFE2-human^cell_line^K562^ENCFF813HVG	0	0.014045358
chr1_20171860_G_A_A	-33	2133	NFE2-human^cell_line^K562^ENCFF813HVG	0	0.021608621
chr1_20171860_G_A_A	-32	2133	NFE2-human^cell_line^K562^ENCFF813HVG	0.002956539	0.011169076
chr1_20171860_G_A_A	-31	2133	NFE2-human^cell_line^K562^ENCFF813HVG	0.003193468	0.010885268
chr1_20171860_G_A_A	-30	2133	NFE2-human^cell_line^K562^ENCFF813HVG	0.006846935	0.010204911
chr1_20171860_G_A_A	-29	2133	NFE2-human^cell_line^K562^ENCFF813HVG	0.011916071	0.003961891
chr1_20171860_G_A_A	-28	2133	NFE2-human^cell_line^K562^ENCFF813HVG	0.023404419	0
chr1_20171860_G_A_A	-27	2133	NFE2-human^cell_line^K562^ENCFF813HVG	0	0.008542389
chr1_20171860_G_A_A	-26	2133	NFE2-human^cell_line^K562^ENCFF813HVG	0.016772181	0
chr1_20171860_G_A_A	-25	2133	NFE2-human^cell_line^K562^ENCFF813HVG	0	0.010730684
chr1_20171860_G_A_A	-24	2133	NFE2-human^cell_line^K562^ENCFF813HVG	0.012056023	0.010449111
chr1_20171860_G_A_A	-23	2133	NFE2-human^cell_line^K562^ENCFF813HVG	0.003410757	0.002555817
chr1_20171860_G_A_A	-22	2133	NFE2-human^cell_line^K562^ENCFF813HVG	0.008947998	0.002692103
chr1_20171860_G_A_A	-21	2133	NFE2-human^cell_line^K562^ENCFF813HVG	0.034406006	0
chr1_20171860_G_A_A	-20	2133	NFE2-human^cell_line^K562^ENCFF813HVG	0.021119922	0
chr1_20171860_G_A_A	-19	2133	NFE2-human^cell_line^K562^ENCFF813HVG	0.041465521	0
chr1_20171860_G_A_A	-18	2133	NFE2-human^cell_line^K562^ENCFF813HVG	0.039732903	0
chr1_20171860_G_A_A	-17	2133	NFE2-human^cell_line^K562^ENCFF813HVG	0.01363337	0
chr1_20171860_G_A_A	-16	2133	NFE2-human^cell_line^K562^ENCFF813HVG	0.019174337	0.012545675
chr1_20171860_G_A_A	-15	2133	NFE2-human^cell_line^K562^ENCFF813HVG	0.018932343	0
chr1_20171860_G_A_A	-14	2133	NFE2-human^cell_line^K562^ENCFF813HVG	0.015577257	0.015765131
chr1_20171860_G_A_A	-13	2133	NFE2-human^cell_line^K562^ENCFF813HVG	0.014862388	0.017767221
chr1_20171860_G_A_A	-12	2133	NFE2-human^cell_line^K562^ENCFF813HVG	0.037336379	0
chr1_20171860_G_A_A	-11	2133	NFE2-human^cell_line^K562^ENCFF813HVG	0.030298144	0.004345864
chr1_20171860_G_A_A	-10	2133	NFE2-human^cell_line^K562^ENCFF813HVG	0.027647048	0.02039808
chr1_20171860_G_A_A	-9	2133	NFE2-human^cell_line^K562^ENCFF813HVG	0	0.053122193
chr1_20171860_G_A_A	-8	2133	NFE2-human^cell_line^K562^ENCFF813HVG	0.090018719	0
chr1_20171860_G_A_A	-7	2133	NFE2-human^cell_line^K562^ENCFF813HVG	0.090978801	0.024758101
chr1_20171860_G_A_A	-6	2133	NFE2-human^cell_line^K562^ENCFF813HVG	0.139202207	0
chr1_20171860_G_A_A	-5	2133	NFE2-human^cell_line^K562^ENCFF813HVG	0.129637361	0
chr1_20171860_G_A_A	-4	2133	NFE2-human^cell_line^K562^ENCFF813HVG	0.143064231	0
chr1_20171860_G_A_A	-3	2133	NFE2-human^cell_line^K562^ENCFF813HVG	0.113156438	0.02865833
chr1_20171860_G_A_A	-2	2133	NFE2-human^cell_line^K562^ENCFF813HVG	0.13452071	0
chr1_20171860_G_A_A	-1	2133	NFE2-human^cell_line^K562^ENCFF813HVG	0.136294365	0
chr1_20171860_G_A_A	**0**	**2133**	**NFE2-human^cell_line^K562^ENCFF813HVG**	**0.146330446**	**0**
chr1_20171860_G_A_A	1	2133	NFE2-human^cell_line^K562^ENCFF813HVG	0.039064229	0.091085821
chr1_20171860_G_A_A	2	2133	NFE2-human^cell_line^K562^ENCFF813HVG	0.09777087	0
chr1_20171860_G_A_A	3	2133	NFE2-human^cell_line^K562^ENCFF813HVG	0.008955628	0
chr1_20171860_G_A_A	4	2133	NFE2-human^cell_line^K562^ENCFF813HVG	0	0.04771027
chr1_20171860_G_A_A	5	2133	NFE2-human^cell_line^K562^ENCFF813HVG	0.023596615	0.04177776
chr1_20171860_G_A_A	6	2133	NFE2-human^cell_line^K562^ENCFF813HVG	0.021921933	0
chr1_20171860_G_A_A	7	2133	NFE2-human^cell_line^K562^ENCFF813HVG	0.016118735	0.024835289
chr1_20171860_G_A_A	8	2133	NFE2-human^cell_line^K562^ENCFF813HVG	0.01158911	0.01297611
chr1_20171860_G_A_A	9	2133	NFE2-human^cell_line^K562^ENCFF813HVG	0.007553488	0.017774254
chr1_20171860_G_A_A	10	2133	NFE2-human^cell_line^K562^ENCFF813HVG	0.003005475	0.031250745
chr1_20171860_G_A_A	11	2133	NFE2-human^cell_line^K562^ENCFF813HVG	0.018148422	0.029260635
chr1_20171860_G_A_A	12	2133	NFE2-human^cell_line^K562^ENCFF813HVG	0	0.012141794
chr1_20171860_G_A_A	13	2133	NFE2-human^cell_line^K562^ENCFF813HVG	0	0.035843849
chr1_20171860_G_A_A	14	2133	NFE2-human^cell_line^K562^ENCFF813HVG	0	0.025410861
chr1_20171860_G_A_A	15	2133	NFE2-human^cell_line^K562^ENCFF813HVG	0.023074031	0.023512185
chr1_20171860_G_A_A	16	2133	NFE2-human^cell_line^K562^ENCFF813HVG	0	0.046833426
chr1_20171860_G_A_A	17	2133	NFE2-human^cell_line^K562^ENCFF813HVG	0	0.017101526
chr1_20171860_G_A_A	18	2133	NFE2-human^cell_line^K562^ENCFF813HVG	0.019244373	0
chr1_20171860_G_A_A	19	2133	NFE2-human^cell_line^K562^ENCFF813HVG	0.011854887	0.007942796
chr1_20171860_G_A_A	20	2133	NFE2-human^cell_line^K562^ENCFF813HVG	0.011339515	0.025097668
chr1_20171860_G_A_A	21	2133	NFE2-human^cell_line^K562^ENCFF813HVG	0.004073173	0.053340524
chr1_20171860_G_A_A	22	2133	NFE2-human^cell_line^K562^ENCFF813HVG	0.002820402	0.010949314
chr1_20171860_G_A_A	23	2133	NFE2-human^cell_line^K562^ENCFF813HVG	0.032688737	0
chr1_20171860_G_A_A	24	2133	NFE2-human^cell_line^K562^ENCFF813HVG	0.003641903	0.013663918
chr1_20171860_G_A_A	25	2133	NFE2-human^cell_line^K562^ENCFF813HVG	0.005484521	0
chr1_20171860_G_A_A	26	2133	NFE2-human^cell_line^K562^ENCFF813HVG	0	0.034766436
chr1_20171860_G_A_A	27	2133	NFE2-human^cell_line^K562^ENCFF813HVG	0.020644933	0.003676981
chr1_20171860_G_A_A	28	2133	NFE2-human^cell_line^K562^ENCFF813HVG	0.004580736	0.025441557
chr1_20171860_G_A_A	29	2133	NFE2-human^cell_line^K562^ENCFF813HVG	0.00807327	0
chr1_20171860_G_A_A	30	2133	NFE2-human^cell_line^K562^ENCFF813HVG	0.024169177	0
chr1_20171860_G_A_A	31	2133	NFE2-human^cell_line^K562^ENCFF813HVG	0.022599638	0
chr1_20171860_G_A_A	32	2133	NFE2-human^cell_line^K562^ENCFF813HVG	0.009856403	0
chr1_20171860_G_A_A	33	2133	NFE2-human^cell_line^K562^ENCFF813HVG	0.026941448	0.005308092
chr1_20171860_G_A_A	34	2133	NFE2-human^cell_line^K562^ENCFF813HVG	0.005417913	0.009893477
chr1_20171860_G_A_A	35	2133	NFE2-human^cell_line^K562^ENCFF813HVG	0.005943656	0.021336287
chr1_20171860_G_A_A	36	2133	NFE2-human^cell_line^K562^ENCFF813HVG	0.006521165	0.013973266
chr1_20171860_G_A_A	37	2133	NFE2-human^cell_line^K562^ENCFF813HVG	0	0.045867354
chr1_20171860_G_A_A	38	2133	NFE2-human^cell_line^K562^ENCFF813HVG	0	0.006804466
chr1_20171860_G_A_A	39	2133	NFE2-human^cell_line^K562^ENCFF813HVG	0.006623715	0.006071925
chr1_20171860_G_A_A	40	2133	NFE2-human^cell_line^K562^ENCFF813HVG	0.002976805	0.005513936

chr1_20171860_G_A_A	41	2133	NFE2-human^cell_line^K562^ENCFF813HVG	0.008466154	0.004719585
chr1_20171860_G_A_A	42	2133	NFE2-human^cell_line^K562^ENCFF813HVG	0.005352199	9.09E-05
chr1_20171860_G_A_A	43	2133	NFE2-human^cell_line^K562^ENCFF813HVG	0.011681408	0
chr1_20171860_G_A_A	44	2133	NFE2-human^cell_line^K562^ENCFF813HVG	0.015129566	0
chr1_20171860_G_A_A	45	2133	NFE2-human^cell_line^K562^ENCFF813HVG	0	0.007766515
chr1_20171860_G_A_A	46	2133	NFE2-human^cell_line^K562^ENCFF813HVG	0.00278011	0.002762645
chr1_20171860_G_A_A	47	2133	NFE2-human^cell_line^K562^ENCFF813HVG	0.012956411	0
chr1_20171860_G_A_A	48	2133	NFE2-human^cell_line^K562^ENCFF813HVG	0	0.003854722
chr1_20171860_G_A_A	49	2133	NFE2-human^cell_line^K562^ENCFF813HVG	0	0.009260207
chr1_20171860_G_A_A	50	2133	NFE2-human^cell_line^K562^ENCFF813HVG	0.006421208	0.002081215
chr1_20171860_G_A_A	51	2133	NFE2-human^cell_line^K562^ENCFF813HVG	0.002903432	0
chr1_20171860_G_A_A	52	2133	NFE2-human^cell_line^K562^ENCFF813HVG	0.000644773	0.008762926
chr1_20171860_G_A_A	53	2133	NFE2-human^cell_line^K562^ENCFF813HVG	0.012990713	0
chr1_20171860_G_A_A	54	2133	NFE2-human^cell_line^K562^ENCFF813HVG	0	0.013671011
chr1_20171860_G_A_A	55	2133	NFE2-human^cell_line^K562^ENCFF813HVG	0.004041374	0.018005699
chr1_20171860_G_A_A	56	2133	NFE2-human^cell_line^K562^ENCFF813HVG	0.001983881	0.005992413
chr1_20171860_G_A_A	57	2133	NFE2-human^cell_line^K562^ENCFF813HVG	0	0.022170633
chr1_20171860_G_A_A	58	2133	NFE2-human^cell_line^K562^ENCFF813HVG	0	0.010280013
chr1_20171860_G_A_A	59	2133	NFE2-human^cell_line^K562^ENCFF813HVG	0	0.009633273
chr1_20171860_G_A_A	60	2133	NFE2-human^cell_line^K562^ENCFF813HVG	0.006203115	0
chr1_20171860_G_A_A	61	2133	NFE2-human^cell_line^K562^ENCFF813HVG	6.29E-05	0.017414778
chr1_20171860_G_A_A	62	2133	NFE2-human^cell_line^K562^ENCFF813HVG	0.003815889	0.001297891
chr1_20171860_G_A_A	63	2133	NFE2-human^cell_line^K562^ENCFF813HVG	0	0.013590395
chr1_20171860_G_A_A	64	2133	NFE2-human^cell_line^K562^ENCFF813HVG	0.000752658	0.006844819
chr1_20171860_G_A_A	65	2133	NFE2-human^cell_line^K562^ENCFF813HVG	0.0107629	0
chr1_20171860_G_A_A	66	2133	NFE2-human^cell_line^K562^ENCFF813HVG	0.003170013	0.002665281
chr1_20171860_G_A_A	67	2133	NFE2-human^cell_line^K562^ENCFF813HVG	0.012526006	0
chr1_20171860_G_A_A	68	2133	NFE2-human^cell_line^K562^ENCFF813HVG	0.000161827	0.002887934
chr1_20171860_G_A_A	69	2133	NFE2-human^cell_line^K562^ENCFF813HVG	0.009975821	0
chr1_20171860_G_A_A	70	2133	NFE2-human^cell_line^K562^ENCFF813HVG	0.00294596	0.001122177
chr1_20171860_G_A_A	71	2133	NFE2-human^cell_line^K562^ENCFF813HVG	0	0.003951579
chr1_20171860_G_A_A	72	2133	NFE2-human^cell_line^K562^ENCFF813HVG	0.010867149	0
chr1_20171860_G_A_A	73	2133	NFE2-human^cell_line^K562^ENCFF813HVG	0.002198845	0
chr1_20171860_G_A_A	74	2133	NFE2-human^cell_line^K562^ENCFF813HVG	0.011283994	0
chr1_20171860_G_A_A	75	2133	NFE2-human^cell_line^K562^ENCFF813HVG	0.002667725	0.000295907
chr1_20171860_G_A_A	76	2133	NFE2-human^cell_line^K562^ENCFF813HVG	0.006802827	0
chr1_20171860_G_A_A	77	2133	NFE2-human^cell_line^K562^ENCFF813HVG	0	0.004703104
chr1_20171860_G_A_A	78	2133	NFE2-human^cell_line^K562^ENCFF813HVG	0.012646556	0
chr1_20171860_G_A_A	79	2133	NFE2-human^cell_line^K562^ENCFF813HVG	0.006464541	0
chr1_20171860_G_A_A	80	2133	NFE2-human^cell_line^K562^ENCFF813HVG	0.008055091	0
chr1_20171860_G_A_A	81	2133	NFE2-human^cell_line^K562^ENCFF813HVG	0.009723127	0
chr1_20171860_G_A_A	82	2133	NFE2-human^cell_line^K562^ENCFF813HVG	0.010518014	0
chr1_20171860_G_A_A	83	2133	NFE2-human^cell_line^K562^ENCFF813HVG	0.011769295	0
chr1_20171860_G_A_A	84	2133	NFE2-human^cell_line^K562^ENCFF813HVG	0.002931297	0.009163588
chr1_20171860_G_A_A	85	2133	NFE2-human^cell_line^K562^ENCFF813HVG	0	0.00949803
chr1_20171860_G_A_A	86	2133	NFE2-human^cell_line^K562^ENCFF813HVG	0.004656911	0
chr1_20171860_G_A_A	87	2133	NFE2-human^cell_line^K562^ENCFF813HVG	0.002456456	0.001152992
chr1_20171860_G_A_A	88	2133	NFE2-human^cell_line^K562^ENCFF813HVG	0.002257705	0.007667035
chr1_20171860_G_A_A	89	2133	NFE2-human^cell_line^K562^ENCFF813HVG	0.002189934	0.005643427
chr1_20171860_G_A_A	90	2133	NFE2-human^cell_line^K562^ENCFF813HVG	0.004996449	0.008992434
chr1_20171860_G_A_A	91	2133	NFE2-human^cell_line^K562^ENCFF813HVG	0.008245021	0.000899971
chr1_20171860_G_A_A	92	2133	NFE2-human^cell_line^K562^ENCFF813HVG	0.009995043	0
chr1_20171860_G_A_A	93	2133	NFE2-human^cell_line^K562^ENCFF813HVG	0.005662441	0.001162261
chr1_20171860_G_A_A	94	2133	NFE2-human^cell_line^K562^ENCFF813HVG	0.002480477	0.002905428
chr1_20171860_G_A_A	95	2133	NFE2-human^cell_line^K562^ENCFF813HVG	0.00043276	0.002353549
chr1_20171860_G_A_A	96	2133	NFE2-human^cell_line^K562^ENCFF813HVG	0.002824962	0.004782081
chr1_20171860_G_A_A	97	2133	NFE2-human^cell_line^K562^ENCFF813HVG	0.000215203	0.006519526
chr1_20171860_G_A_A	98	2133	NFE2-human^cell_line^K562^ENCFF813HVG	0.003863811	0.001553148
chr1_20171860_G_A_A	99	2133	NFE2-human^cell_line^K562^ENCFF813HVG	0.00324145	0.00220561
chr1_20171860_G_A_A	100	2133	NFE2-human^cell_line^K562^ENCFF813HVG	0.010320395	0

The table contains 6 columns.

The details are:

Variant: sequences contain reference allele or alternative allele.
Position: from variant's upstream 99 (-99) to downstream 100 bp.
Profile_index: index of chromatin profile in DeepFun model.
Profile_name: name of chromatin profile in DeepFun model.
Max_loss: the max gained SAD value by *in silico* saturated mutagenesis analysis.
Max_gain: the max lost SAD value by *in silico* saturated mutagenesis analysis.
